# A mechanical G2 checkpoint controls epithelial cell division through E-cadherin-mediated regulation of Wee1-Cdk1

**DOI:** 10.1016/j.celrep.2022.111475

**Published:** 2022-10-11

**Authors:** Lisa Donker, Ronja Houtekamer, Marjolein Vliem, François Sipieter, Helena Canever, Manuel Gómez-González, Miquel Bosch-Padrós, Willem-Jan Pannekoek, Xavier Trepat, Nicolas Borghi, Martijn Gloerich

**Affiliations:** 1 Center for Molecular Medicine, University Medical Center Utrecht, Universiteitsweg 100, 3584 Utrecht, the Netherlands; 2 Université Paris Cité, CNRS, Institut Jacques Monod, 75013 Paris, France; 3 Institute for Bioengineering of Catalonia (IBEC), Barcelona Institute for Science and Technology (BIST), 08028 Barcelona, Spain; 4 Facultat de Medicina, Universitat de Barcelona, 08036 Barcelona, Spain; 5 Institució Catalana de Recerca i Estudis Avançats (ICREA), Barcelona, Spain; 6 Centro de Investigación Biomédica en Red en Bioingeniería, Biomateriales y Nanomedicina (CIBER-BBN), 08028 Barcelona, Spain

## Abstract

Epithelial cell divisions are coordinated with cell loss to preserve epithelial integrity. However, how epithelia adapt their rate of cell division to changes in cell number, for instance during homeostatic turnover or wounding, is not well understood. Here, we show that epithelial cells sense local cell density through mechanosensitive E-cadherin adhesions to control G2/M cell-cycle progression. As local cell density increases, tensile forces on E-cadherin adhesions are reduced, which prompts the accumulation of the G2 checkpoint kinase Wee1 and downstream inhibitory phosphorylation of Cdk1. Consequently, dense epithelia contain a pool of cells that are temporarily halted in G2 phase. These cells are readily triggered to divide following epithelial wounding due to the consequent increase in intercellular forces and resulting degradation of Wee1. Our data collectively show that epithelial cell division is controlled by a mechanical G2 checkpoint, which is regulated by cell-density-dependent intercellular forces sensed and transduced by E-cadherin adhesions.

## Graphical abstract

**Graphical abstract:**
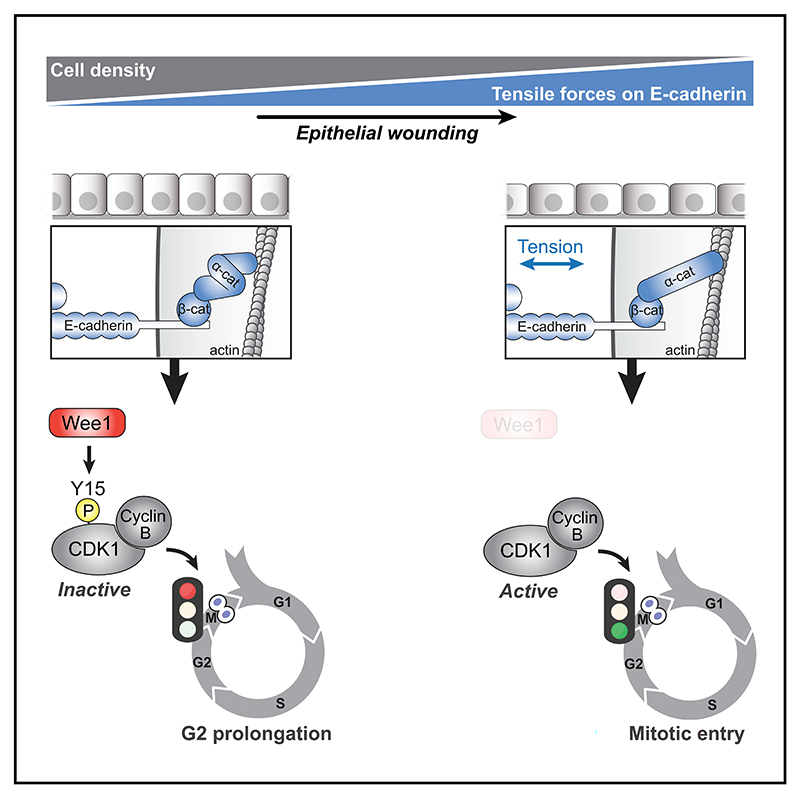

## Introduction

Epithelia form protective barriers covering the body surface and internal organs while being dynamic with high rates of cellular turnover. To preserve the integrity of the epithelial barrier and at the same time prevent tissue overgrowth, epithelial cell division must be tightly coordinated with cell loss. This requires epithelia to respond to fluctuations in cell number, for instance during homeostatic turnover or wounding, and adjust their rate of cell division accordingly. Disruption of this proliferative response underlies epithelial barrier disorders (Eming et al., 2014) and epithelial cancers in which density-dependent inhibition of proliferation is lost (McClatchey and Yap, 2012). Understanding how epithelial homeostasis is achieved and maintained requires identification of the mechanisms by which epithelia sense variations in cell number and transmit this information to adapt division rate.

Epithelial cell division can be induced by activation of quiescent cells that have exited the cell cycle, and it has previously been demonstrated that cells residing in G0/G1 are triggered to re-enter the cell cycle following wounding of the epithelial layer (Streichan et al., 2014; Bornes et al., 2021). In most epithelial tissues, however, cells with proliferative capacity are already actively cycling during homeostasis to drive tissue self-renewal (Li and Clevers, 2010). The division rate of these cells was long presumed to be independent of external regulation, as it has been a prevailing dogma that the cell cycle becomes irresponsive to extrinsic cues following G1/S transition (Pardee, 1989). However, findings in invertebrate model organisms revealed that progression through later cell-cycle stages can be extrinsically regulated, for instance by nutrient signaling (Otsuki and Brand, 2018; Seidel and Kimble, 2015; McKeown and Cline, 2019). Yet, how such extrinsic signals influence progression through later stages of the cell cycle, and thereby the rate of cell division in epithelia, is not well understood.

Extrinsic regulation of cell proliferation involves biochemical signals (e.g., nutrients and growth factors) but also mechanical forces originating from neighboring cells and the extracellular environment (LeGoff and Lecuit, 2016; Godard and Heisenberg, 2019). Cells possess a repertoire of mechanosensor proteins that can transduce these mechanical cues into an intracellular response, involving force-dependent changes in their conformation and sub-sequent induction of downstream signaling pathways (Swaminathan and Gloerich, 2021). As such, mechanotransduction by cadherin- and integrin-based adhesions that anchor cells to each other and the surrounding tissue, respectively, contribute to the regulation of cell-cycle exit and re-entry (Aragona et al., 2013; Benham-Pyle et al., 2015; Rauskolb et al., 2014; Dutta et al., 2018; Ibar et al., 2018). The force-sensitive calcium channel Piezo1 is also linked to epithelial cell proliferation and was recently shown to regulate mitotic entry following ectopic application of mechanical stretch (Gudipaty et al., 2017). Nonetheless, the forces that cells experience during epithelial homeostasis, and how these forces regulate cell-cycle progression, remain poorly understood.

Here, we show that epithelial cells sense changes in cell number through coinciding changes in intercellular forces, which are transduced by E-cadherin adhesions to control a cell-density-dependent G2 checkpoint. This checkpoint establishes a pool of cells in dense epithelia that are halted in G2 phase, mediated by force-sensitive regulation of the kinase Wee1 that maintains the mitotic gatekeeper Cdk1 in an inactive state. Increased tension on E-cadherin adhesions, which occurs for instance during epithelial expansion upon wounding, triggers rapid degradation of Wee1 and subsequent mitotic entry of G2-halted cells. Our data thus demonstrate that epithelia coordinate cell divisions with local cell density through a mechanical G2 checkpoint regulated by E-cadherin mechanotransduction.

## Results

### Rapid induction of mitotic entry during epithelial expansion following wounding

**Figure 1:**
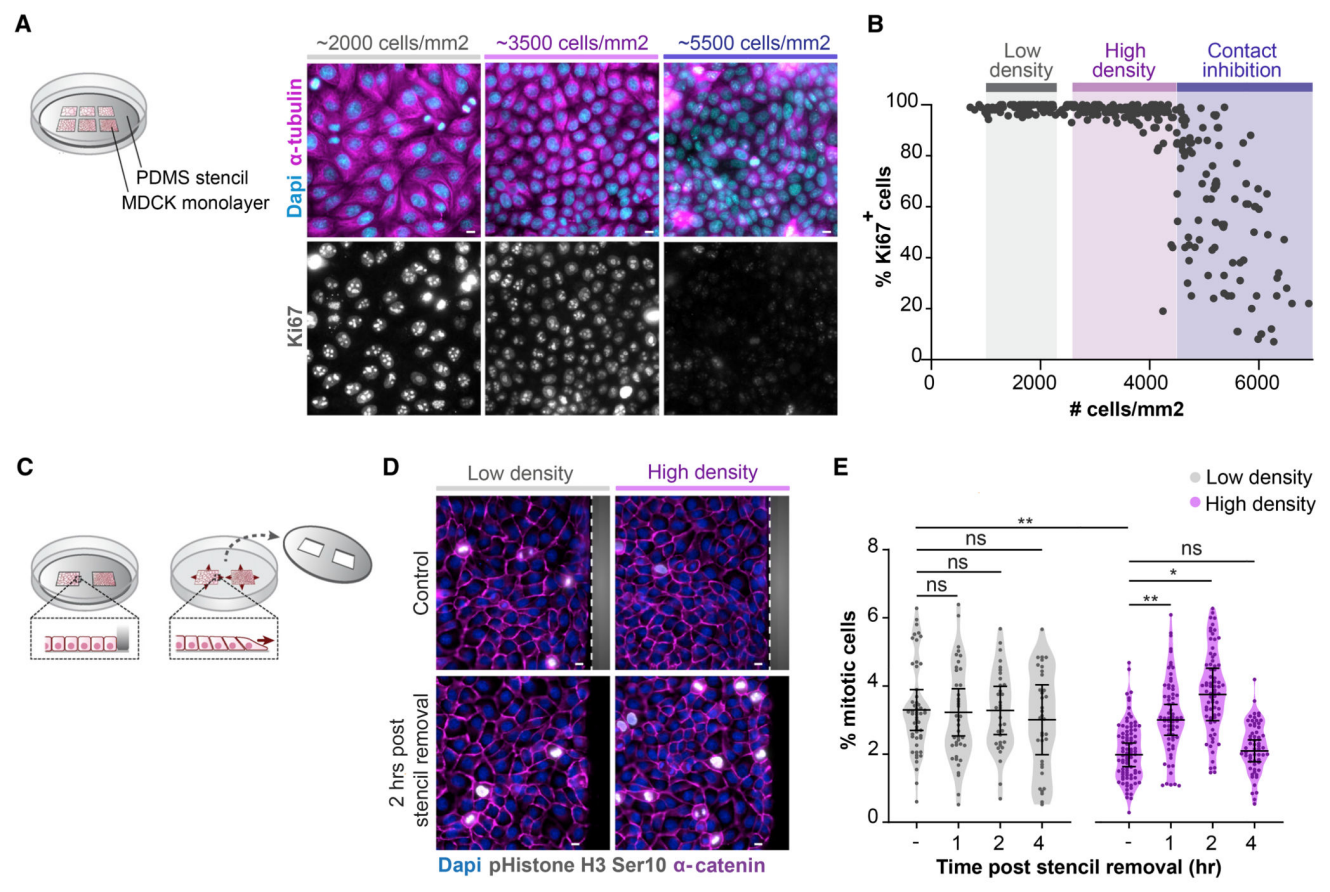
Rapid induction of mitotic entry during epithelial expansion following wounding (A) Left: schematic image of wells within a PDMS stencil that is placed on a collagen I-coated glass surface, with different densities of MDCK cells plated in each well. All monolayers are exposed to the same culture medium and thus identical concentrations of nutrients and growth factors. Right: immunostaining of MDCK monolayers at various cell densities for Ki67 together with α-tubulin and Dapi. (B) Quantification of the percentage of Ki67^+^ cells in MDCK monolayers grown at various cell densities. The range of densities considered low-density monolayers (1,000–2,200 cells/mm^2^), high-density monolayers (2,600–4,500 cells/mm^2^), and contact-inhibited density (CIP) (>4,500 cells/mm^2^) are indicated in gray, magenta, and blue. Data were pooled from 3 independent experiments, with >50 monolayer regions per experiment. (C) Schematic image of the stencil removal assay to mimic epithelial expansion following wounding. (D) Immunostaining of MDCK monolayers grown at low and high density (see Figure 1B) without and 2 h after removal of the PDMS stencil, for pS10 histone H3 together with α-catenin and Dapi. The PDMS stencil is indicated in gray, and the dotted line indicates the border between the stencil and cell monolayer. (E) Quantification of the percentage of mitotic cells in MDCK monolayers grown at low (gray) and high (magenta) monolayer density before and 1, 2, and 4 h post stencil removal. n > 30 monolayer regions per condition. Data were pooled from 4 independent experiments. Black bars represent the mean and SD of the individual experiments. *p = 0.023; **p < 0.008, ns, not significant; paired t test. All scale bars represent 10 μm. See also Figure S1.

To investigate how epithelial cell divisions are coordinated with cell loss, we analyzed cell division rate following wounding of monolayers of Madin-Darby canine kidney (MDCK) cells that recapitulate the organization of a single-layered epithelial tissue (Mays et al., 1995). Characterization of MDCK monolayers 24 h after plating in wells within silicone (polydimethylsiloxane [PDMS]) stencils demonstrated that they formed confluent monolayers starting at ~1,000 cells/mm^2^ and remained almost fully Ki67-positive and thus actively cycling up to a density of ~4,500 cells/mm (Figures 1A and 1B). We therefore defined MDCK monolayers of 2,600–4,500 cells/mm^2^ as high-density (yet still actively cycling) monolayers, 1,000–2,200 cells/mm^2^ as low-density monolayers, and >4,500 cells/mm^2^ as monolayers undergoing contact inhibition of proliferation and exiting the cell cycle. Next, we mimicked epithelial wounding by removal of the PDMS stencil (Figure 1C), which allows the monolayer to freely expand into the newly available space (Poujade et al., 2007). This approach recapitulates the changes in cell motility and concurrent epithelial expansion that occur upon epithelial wounding in the absence of cell damage induced by traditional wounding assays (Poujade et al., 2007). Strikingly, in monolayers cultured at high density, we observed a burst of mitotic events within 1–2 h after stencil removal (Figures 1D and 1E). This increase in mitotic cells was transient and returned to basal levels within 4 h (Figure 1E). In low-density monolayers, the basal level of mitotic events was higher compared with dense monolayers but remained unaltered following stencil removal (Figures 1D and 1E). Expectedly, contact-inhibited monolayers (>4,500 cells/mm^2^) also did not show an elevation of mitotic events within the first hours following stencil removal (Figure S1A). These data indicate that dense monolayers of actively cycling cells contain a pool of cells that is rapidly induced to enter mitosis and divide during epithelial expansion following wounding.

### G2 phase of the cell cycle is prolonged in dense epithelial monolayers

**Figure 2:**
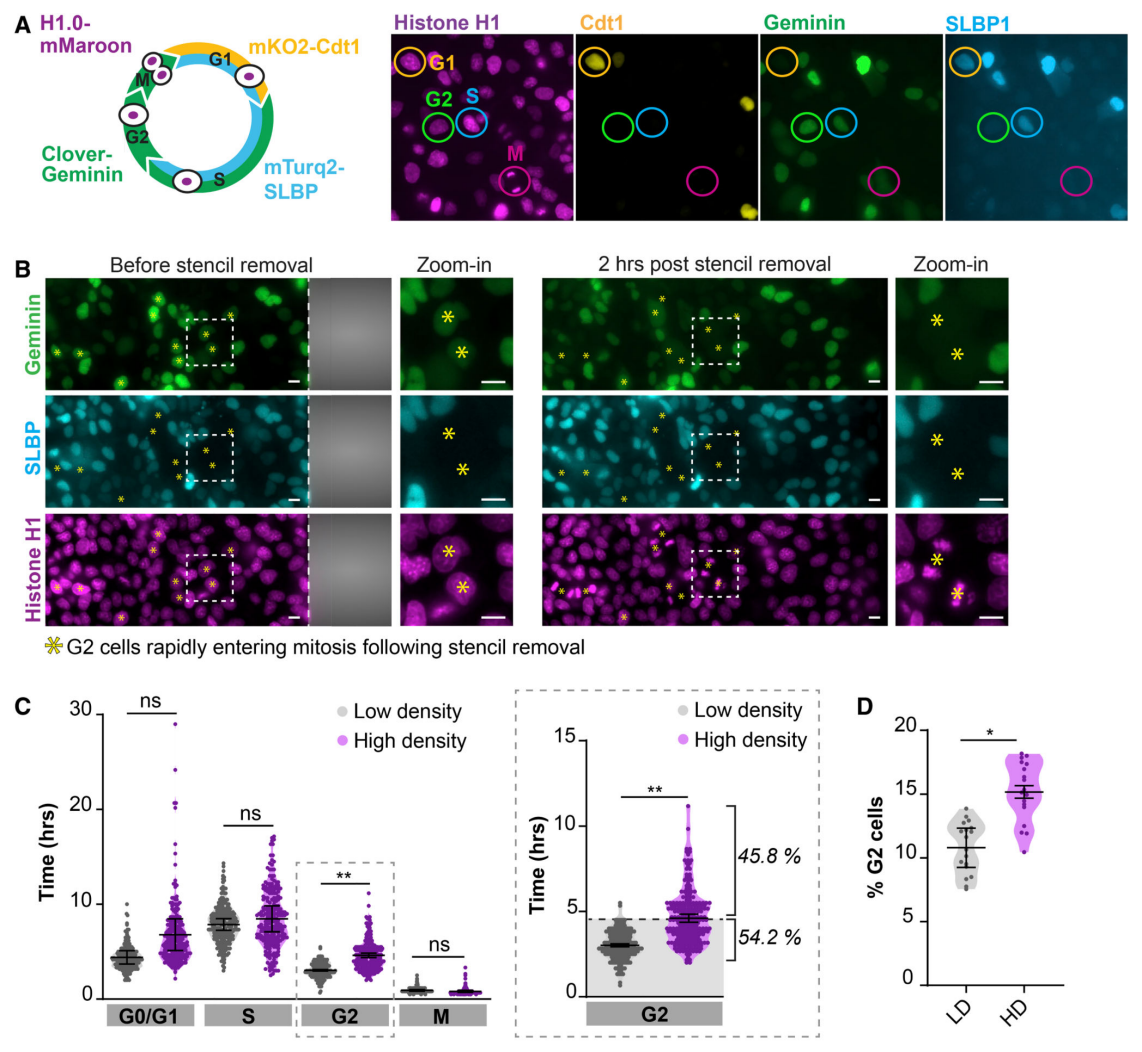
Prolongation of G2 phase in dense epithelial monolayers (A) Schematic image of the FUCCI4 reporter (Bajar et al., 2016) with differential expression of mKO-Cdt1(30–120), mTurquoise2-SLBP(18–126), and Clover-Geminin(1–110) throughout the cell cycle, together with H1.0-mMaroon to visualize chromosome condensation in mitosis. (B) Visualization of cell-cycle stages using the FUCCI4 reporter in dense MDCK monolayers before and 2 h after removal of the PDMS stencil. 99 out of 100 cells that entered mitosis within 2.5 h following stencil removal (asterisk) were in G2 phase prior to stencil removal (indicated by expression of Clover-Geminin(1–110) and absence of mTurquoise2-SLBP(18–126)). One cell resided in the end of S phase and quickly proceeded through G2 following stencil removal. Scale bars represent 10 μm. (C) Left: quantification of the duration (h) of the different cell-cycle phases of cells grown at low (gray) and high (magenta) density. Note that G0 and G1 phases cannot be distinguished by the FUCCI4 reporter, although at the cell densities used in this experiment, very few cells are in G0 (Figure 1B). While the average length of G1 phase does not significantly change (p = 0.1467), there is a pool of cells (22.2% ± 16.07%) at high density with a prolonged (>8 h) G1 phase (see Figure S1C). The inset shows the duration of G2 phase (h), with the 99% percentile of the duration of G2 in low-density monolayers indicated (4.5 h, dashed line). 45.8% ± 6.2% of cells in high-density monolayers show a prolonged G2 phase of more than 4.5 h. n = 225 cells per condition. Data were pooled from 3 independent experiments. Black bars represent the mean and SD of the individual experiments. **p < 0.0034; ns, not significant; paired t test. (D) Quantification of the percentage of G2 phase cells in low- and high-density monolayers. n = 18 monolayer regions per condition. Data were pooled from 3 independent experiments. Black bars represent the mean and SD of the individual experiments. *p = 0.0275; paired t test. See also Figure S1.

As the mitotic response during wounding-induced expansion of dense epithelia occurred within 1–2 h, this suggests that the cells triggered to enter mitosis were in the G2 phase of the cell cycle. Indeed, visualization of all cell-cycle phases by stable expression of the FUCCI4 reporter (Figure 2A; Bajar et al., 2016) showed that virtually all (99 out of 100) cells entering mitosis during early expansion of dense monolayers resided in G2 prior to stencil removal (Figure 2B). Because the mitotic response following stencil removal occurred only in dense monolayers (Figures 1D and 1E), we hypothesized that these contain a larger population of G2 cells that are able to respond to wounding compared with low-density monolayers. To investigate this, we determined the length of G2 and other cell-cycle phases in MDCK-FUCCI4 monolayers. In low-density monolayers, the average duration of G2 phase was 3.0 ± 0.8 h, with 99% of the cells completing G2 phase within 4.5 h (Figure 2C). At high cell density, the duration of G2 phase was significantly prolonged (Figure 2C) and strongly anti-correlated with the local distance between cell nuclei (Figure S1B). While the average increase in G2 length at high density was ~1.6 h (from 3.0 ± 0.8 to 4.6 ± 1.5 h), a significant population of cells (45.8% ± 6.2%) displayed a prolonged G2 phase, ranging from 4.5 up to ~10 h (Figure 2C).

The average duration of the G0/G1 phases (which cannot be separated from each other by FUCCI4) and S phase did not significantly differ between both monolayer densities (Figure 2C). However, high-density monolayers did contain a substantial number of cells (22.2% ± 16.07%) with increased G0/G1 length compared with low-density monolayers (Figures 2C and S1C). We did not observe a clear correlation between the length of G0/G1 and G2 phases within individual cells (Figure S1D), and the majority of cells with a prolonged (>4.5 h) G2 phase (65.9% ± 20.4%) did not show a similar G0/G1 prolongation earlier in their cell cycle (Figure S1E). This suggests that although both G0/G1 and G2 lengths are increased at high cell density, the underlying mechanisms by which cell density influences these cell-cycle phases are likely distinct.

As a result of the G2 prolongation, dense epithelia contained a significantly increased number of cells in G2 (average of 15.2%) compared with low-density monolayers (average of 10.8%) (Figure 2D). To exclude that the extended G2 length and concurrent increase in the pool of G2 cells at high density is a consequence of increased DNA damage, we assessed the amount of DNA double-strand breaks by immunostaining for γH2AX (Figure S1F). This showed the absence of γH2AX foci at high density, which, in contrast, were clearly present following UV irradiation, indicating that the density-dependent G2 regulation occurs independently of a DNA-damage response.

Taken together, our data indicate that in epithelial layers, the duration of G2 phase is influenced by local cell density. This results in a larger pool of G2 cells in dense epithelia that can be rapidly triggered to enter mitosis during epithelial expansion following wounding.

### Density-dependent G2/M regulation requires mechanotransduction through E-cadherin adhesions

**Figure 3:**
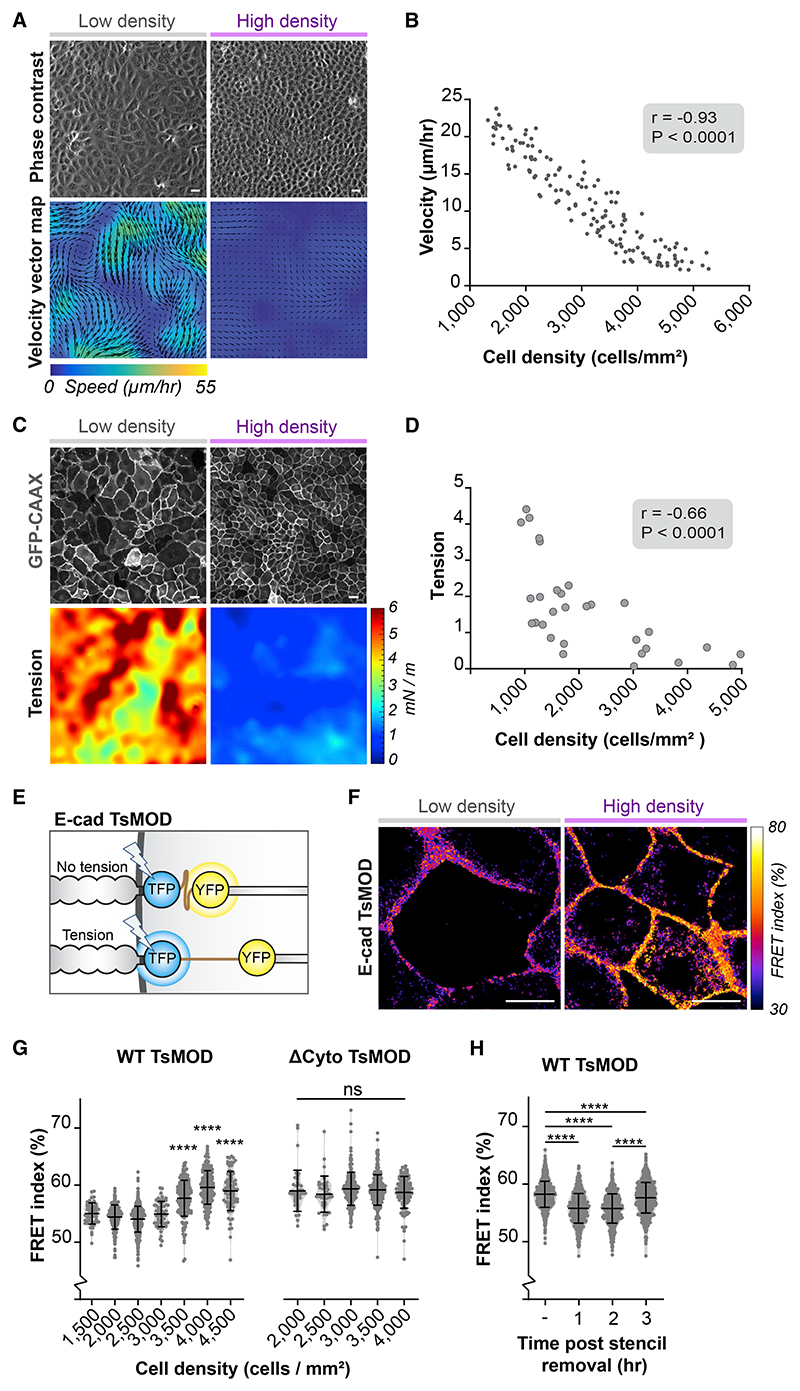
E-cadherin adhesions mechanically sense variations in cell density (A) Phase contrast images of MDCK monolayers grown at low and high density with corresponding vector magnitude heatmaps of cell motility quantified by particle image velocimetry (PIV) analysis. The direction and length of the arrows indicate the direction and speed of motility. Scale bars represent 20 μm. (B) Quantification of cell motility (μm/h) at various MDCK monolayer densities. n = 150; data were pooled from 3 independent experiments. r = −0.93, p < 0.0001; Pearson correlation. (C) Representative examples of MDCK monolayers expressing GFP-CAAX at low and high density with maps of monolayer tension (tr(σ) · h [mN/m]) (corresponding traction force maps are shown in Figure S2A). Scale bars represent 20 μm. (D) Quantification of average normal monolayer tension (tr(σ) · h [mN/m]) at various MDCK monolayer densities. n = 30; data were pooled from 7 independent experiments. r = −0.66, p < 0.0001; Pearson correlation (corresponding traction forces are shown in Figure S2B). (E) Schematic representation of the FRET-based E-cadherin tension sensor (E-cadherin TsMod), in which a mTFP1/YFP FRET pair separated by a flexible linker is placed in the E-cadherin cytosolic tail. (F) Color-coded representation of the FRET index of E-cadherin TsMod in MDCK cells grown at low (left) or high (right) monolayer density. Scale bars represent 10 μm. (G) Graphs showing the FRET index (%) of individual cell-cell contacts of cells expressing E-cadherin TsMod (left) or the tension-insensitive control sensor E-cadherin–ΔCyto TsMod (right) grown at various cell densities. The indicated cell densities each represent a small range of densities ±250 cells/mm^2^. Data were pooled from two independent experiments. Black bars represent the mean and SD. ****p < 0.0001; ns, not significant; Mann-Whitney. (H) Graph showing the FRET index (%) of individual cell-cell contacts of cells expressing E-cadherin TsMod (grown at high monolayer density) before and at indicated time points post stencil removal. Data were pooled from two independent experiments. Black bars represent the mean and SD. ****p < 0.0001; Mann-Whitney. Of note, whereas high-density monolayers show a transient elevation of tension on E-cadherin following stencil removal, monolayers at lower density (with higher basal levels of tension) showed a transient decrease in tension following stencil removal (data not shown and Gayrard et al., 2018). See also Figure S2.

We next investigated how cells sense differences in monolayer density to adapt G2 length. As epithelial monolayers increase in cell density, the motility of epithelial cells gradually declines as visualized by particle image velocimetry (PIV) analysis (Figures 3A and 3B; Petitjean et al., 2010). This gradual decrease in motility occurred across densities at which we observed G2 prolongation and plateaued at a density of ~4,500 cells/mm^2^ (Figure 3B). The reduced cellular movements in dense monolayers could potentially impact the level of forces that cells exert on each other. To map these intercellular forces, we performed monolayer stress microscopy (MSM; Tambe et al., 2011) of monolayers at various densities. This showed a strong reduction in intercellular tension as cells reach high density (Figures 3C and 3D), in line with the density-dependent reduction of cell motility (Figures 3A and 3B).

Intercellular forces can be sensed and transduced by E-cadherin adhesions, which form homotypic interactions between neighboring cells and are linked to the actin cytoskeleton through associated catenin proteins (Leckband and de Rooij, 2014). To test whether an increase in cell density results in a reduction in tension on the E-cadherin complex, we made use of an E-cadherin tension sensor (E-cadherin TsMod), in which tensile forces separate the mTFP1/YFP fluorescence resonance energy transfer (FRET) pair placed in the cytosolic tail of E-cadherin (Figure 3E; Borghi et al., 2012). We observed a gradual increase in FRET ratios corresponding to a reduction in tension on E-cadherin as monolayer density increases above 3,000 cells/mm^2^ and plateauing at a density of ~4,000 cells/mm^2^ (Figures 3F, 3G, and S2C). Importantly, this density-dependent increase in FRET level was not observed with a force-insensitive sensor that is not linked to the actin cytoskeleton, as it lacks part of the E-cadherin cytosolic tail (E-cadherin TsMod ΔCyto) (Figures 3G and S2C). Upon removal of the PDMS stencil, dense epithelia showed a transient decrease in E-cadherin TsMod FRET ratio (Figure 3H). This indicates an elevation of intercellular forces on E-cadherin adhesions during epithelial expansion following removal of a physical constraint, in line with previous MSM analysis of expanding epithelial monolayers (Serra-Picamal et al., 2012). Thus, our data show that changes in G2 length coincide with variations in tensile forces on E-cadherin adhesions between neighboring cells, with low tension on E-cadherin in dense monolayers coinciding with G2 prolongation and increased tension following stencil removal with induction of mitosis.

**Figure 4:**
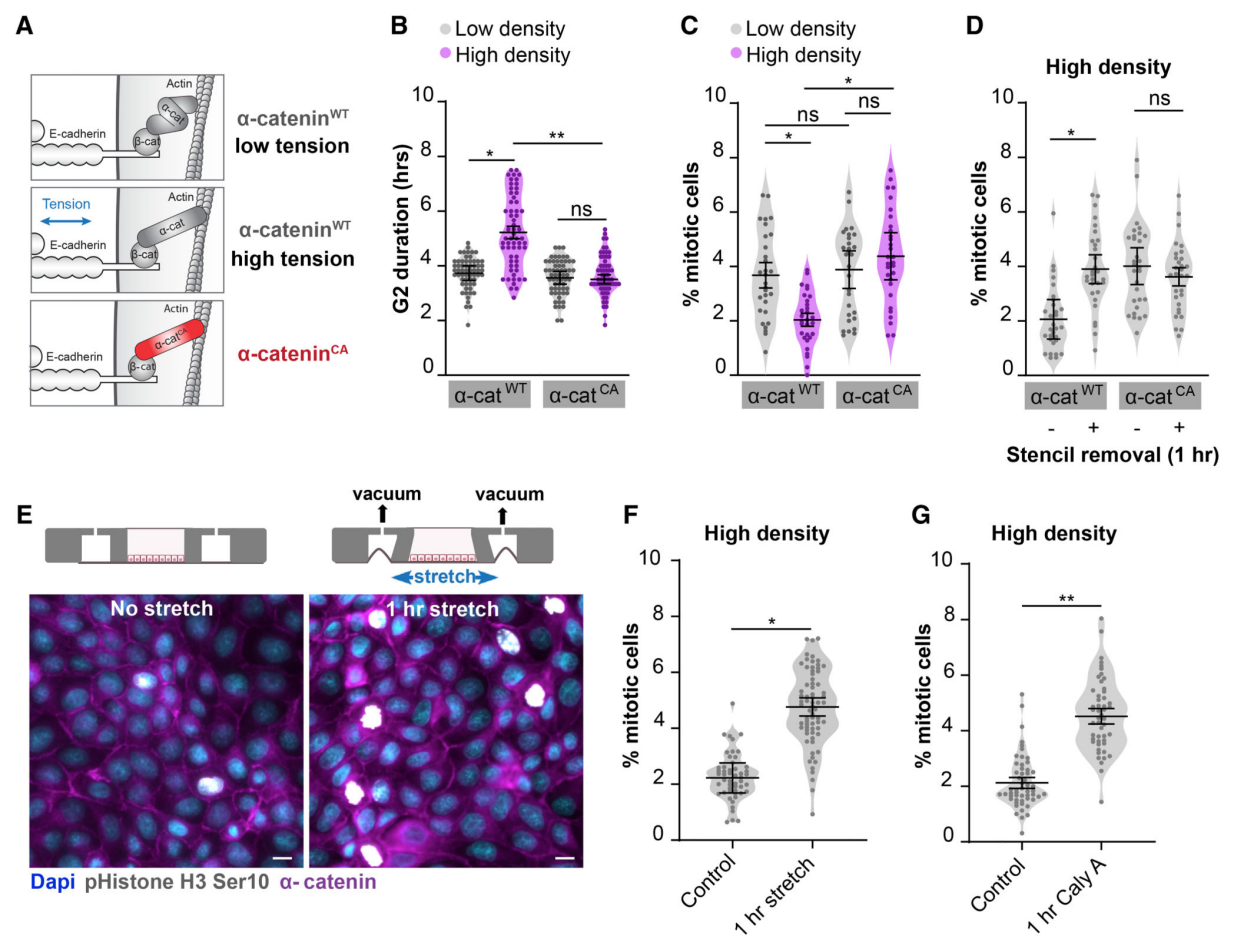
Density-dependent G2/M regulation requires mechanotransduction through E-cadherin adhesions (A) Schematic representation of conformationally active α-catenin (α-catenin^CA^; M319G, R326E α-catenin). Tension on the E-cadherin complex results in unfolding of α-catenin, which is mimicked by expression of the α-catenin^CA^ mutant that is constitutively in an open conformation irrespective of changes in intercellular tension. (B) Quantification of the duration of G2 length (h), based on mTurquoise-SLBP(18–126) expression, in cells expressing either WT or constitutively open (CA) α-catenin-mCherry at low (gray) and high (magenta) monolayer density. n = 60 cells per condition. Data were pooled from 3 independent experiments. Black bars represent the mean and SD of the individual experiments. *p = 0.0116; **p = 0.0079; ns, not significant; paired t test. (C) Quantification of the percentage of mitotic cells in MDCK monolayers expressing either WT or CA α-catenin-mCherry, grown at low (gray) or high (magenta) density. n = 30 fields of view per condition. Data were pooled from 3 independent experiments. Black bars represent the mean and SD of the individual experiments. *p = 0.031 (WT low density versus WT high density), *p = 0.03 (WT high density versus CA high density), ns, not significant; paired t test. (D) Quantification of the percentage of mitotic cells in MDCK monolayers expressing either WT or CA α-catenin-mCherry grown at high density, before and 1 h post stencil removal. n = 30 fields of view per condition. Data were pooled from 3 independent experiments. Black bars represent the mean and SD of the individual experiments. *p = 0.02, ns, not significant; paired t test. (E) Representative image of dense MDCK monolayers, with or without application of 18% uniaxial stretch (1 h), in which mitotic cells are visualized by immunostaining for phospho-histone H3 Ser10 together with α-catenin and Dapi. Scale bars represent 10 μm. (F) Quantification of the percentage of mitotic cells in dense MDCK monolayers with or without application of 18% uniaxial stretch for 1 h. n > 50 monolayer regions per condition. Data were pooled from 3 independent experiments. Black bars represent the mean and SD of the individual experiments. *p = 0.0229; paired t test. (G) Quantification of the percentage of mitotic cells in high-density MDCK monolayers with or without 1 h treatment with Calyculin A (10 ng/mL). n > 50 monolayer regions per condition. Data were pooled from 3 independent experiments. Black bars represent the mean and SD of the individual experiments. **p = 0.0023; paired t test. See also Figure S2.

To test if changes in tension on E-cadherin adhesions are responsible for the density-dependent G2 regulation, we manipulated the mechanism by which E-cadherin adhesions transduce forces. Tensile forces induce a conformational opening of the cadherin-complex component α-catenin, which results in the force-sensitive recruitment of additional proteins to the cadherin complex that can establish downstream signaling (Yonemura et al., 2010; Rauskolb et al., 2014). To render cadherin adhesions insensitive to variations in intercellular forces, we ectopically expressed an α-catenin mutant that is constitutively in an open conformation and thereby mimics its tensed state irrespective of the level of intercellular forces (M319G, R326E α-catenin; constitutively open α-catenin [α-catenin^CA^]; Figures 4A, S2D, and S2E) (Maki et al., 2016; Matsuzawa et al., 2018). At low density, cells expressing either mCherry-tagged wild-type α-catenin (α-catenin^WT^) or α-catenin^CA^ showed similar G2 lengths (Figure 4B). However, whereas α-catenin^WT^ cells displayed an increased G2 length at high density, this G2 prolongation was not observed in dense monolayers of α-catenin^CA^ cells (Figure 4B). As expected from this attenuated density-dependent G2 prolongation in α-catenin^CA^ cells, both the reduction of mitotic rate at high cell density as well as the induction of mitotic entry by stencil removal were lost in these cells (Figures 4C, 4D, and S2F). These data indicate that the increase in G2 length at high density results from a decrease in mechanical tension sensed and transduced by E-cadherin adhesions.

Next, we directly tested whether an acute increase of intercellular forces, as occurs following epithelial wounding, can trigger G2 cells to enter mitosis. For this, we artificially increased mechanical tension by application of uniaxial stretch to dense monolayers (Figure 4E). We observed a rapid burst of mitotic events after 1 h of mechanical stretch (Figures 4E and 4F), similar to the response following stencil removal (Figures 1D and 1E) and in line with recent findings in epithelial cultures and *Xenopus* embryos (Nestor-Bergmann et al., 2019; Gudipaty et al., 2017). Elevating tension in dense monolayers through myosin II-generated contractility, by incubation with the myosin-light chain phosphatase inhibitor Calyculin A (Acharya et al., 2018), resulted in a comparable induction of mitotic events within 1 h (Figure 4G). These data indicate that increasing mechanical tension relieves the G2 prolongation in dense epithelia and triggers rapid mitotic entry.

### An E-cadherin mechanoresponse establishes density-dependent regulation of Wee1 levels

**Figure 5:**
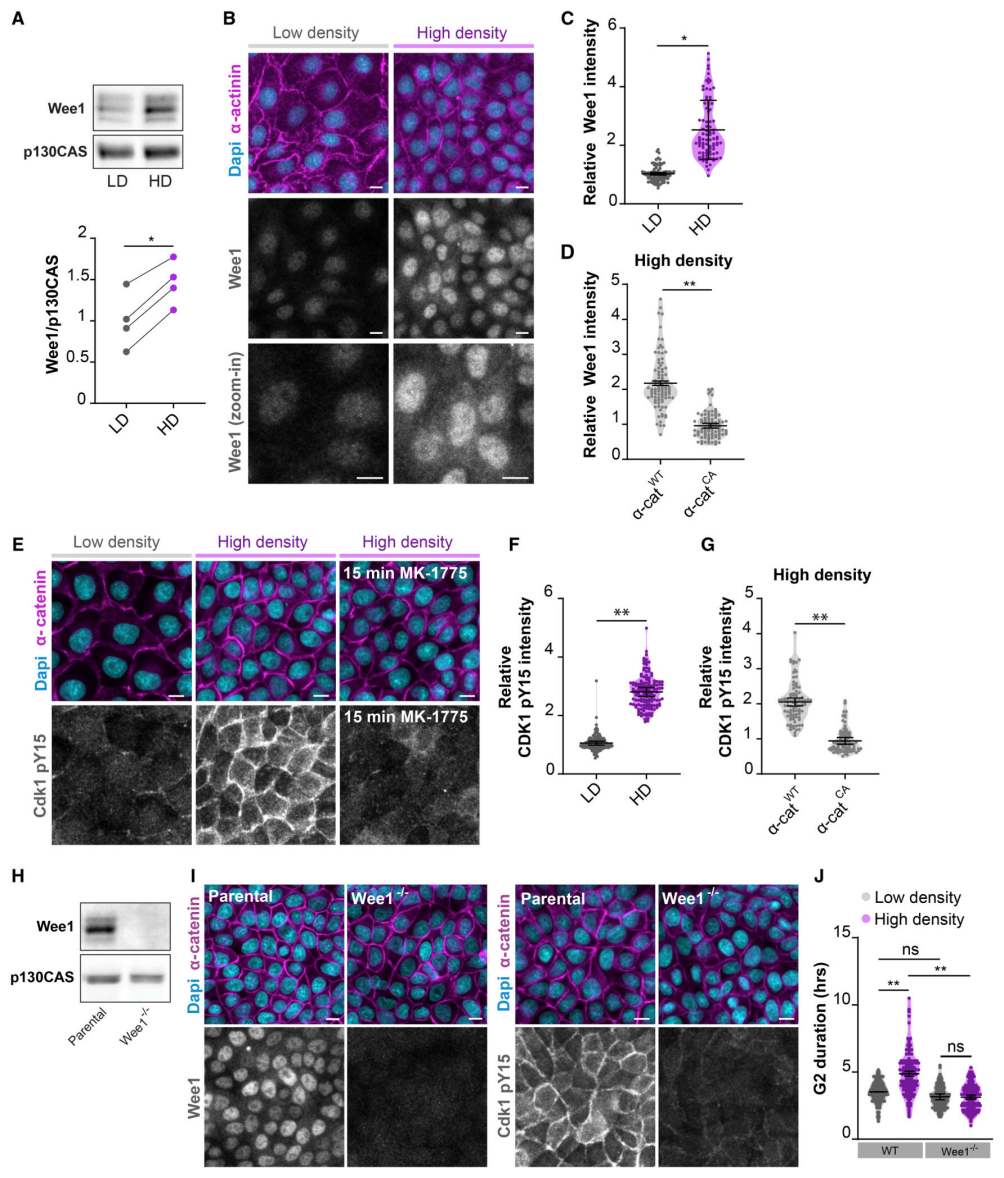
An E-cadherin mechanoresponse establishes density-dependent regulation of Wee1 levels (A) Top: western blot of lysates from MDCK cells grown at low and high monolayer density (LD and HD, respectively) probed for Wee1 and p130CAS. Bottom: quantification of the ratio of Wee1 to p130CAS of 4 independent experiments. *p = 0.0143; ratio paired t test. (B) MDCK monolayers grown at LD and HD and immunostained for Wee1 and α-actinin, together with Dapi. (C) Quantification of Wee1 immunofluorescence intensity per cell (normalized to the level of α-actinin) in MDCK cells grown at low and high monolayer density. n = 90 cells per condition. Data were pooled from 3 independent experiments. Black bars represent the mean and SD of the individual experiments. *p = 0.048; ratio paired t test. (D) Quantification of Wee1 immunofluorescence intensity per cell (normalized to the level of β-catenin intensity) in MDCK cells expressing either WT or CA α-catenin-mCherry. n = 90 cells per condition. Data were pooled from 3 independent experiments. Black bars represent the mean and SD of the individual experiments. **p = 0.0014; ratio paired t test. (E) Immunostainings for Cdk1 pY15 and α-catenin, together with Dapi, in MDCK monolayers grown at LD and HD and at HD after 15 min treatment with the Wee1 inhibitor MK-1775 (500 nM). (F) Quantification of Cdk1 pY15 immunofluorescence intensity per cell (normalized to the level of α-catenin intensity) in MDCK cells grown at LD and HD. n = 150 cells per condition. Data were pooled from 3 independent experiments. Black bars represent the mean and SD of the individual experiments. **p = 0.0031; ratio paired t test. (G) Quantification of Cdk1 pY15 immunofluorescence intensity per cell (normalized to the level of β-catenin intensity) in HD MDCK monolayers expressing either WT or CA α-catenin-mCherry. n = 90 cells per condition. Data were pooled from 3 independent experiments. Black bars represent the mean and SD of the individual experiments. **p = 0.0013; ratio paired t test. (H) Western blot of lysates from parental and *Wee1*^–/–^ MDCK cells (with guide sequence 1, clone g1) probed for Wee1 and p130CAS. The uncropped version of this western blot, also including lysates from *Wee1*^–/–^ MDCK cells with guide sequence 2 (clone g2), is included in Figure S4A. (I) Immunostaining of parental and *Wee1*^–/–^ MDCK cells (clone g1) for Wee1 (left) and Cdk1 pY15 (right), together with α-catenin and Dapi. (J) Quantification of the duration of G2 phase, based on expression of mTurquoise-SLBP(18–126), in parental MDCK and *Wee1*^–/–^ MDCK cells (clone g1) grown at low and high monolayer density. n = 120 cells per condition. Data were pooled from 3 independent experiments. Black bars represent the mean and SD of the individual experiments. **p = 0.0071 (LD WT versus HD WT), **p = 0.0024 (HD WT versus HD *Wee1*^–/–^ guide 1). Quantification of G2 duration in *Wee1*^–/–^ clone g2 cells is shown in Figure S4C. All scale bars represent 10 μm. See also Figures S3 and S4.

Next, we aimed to identify the molecular components downstream of E-cadherin that control the density-dependent G2/M transition. We analyzed potential changes in expression patterns of well-known regulators of G2/M progression between low- and high-density monolayers by western blot analysis and observed in dense monolayers a strong upregulation of Wee1 (Figure 5A), a central kinase in the regulation of G2/M transition (Ghelli Luserna Di Rorà et al., 2020; Koh, 2022). Analyses of Wee1 by immunostaining validated the increase in Wee1 levels at high monolayer density, with Wee1 accumulating both in the nucleus and cytosol (Figures 5B and 5C). Importantly, expression of α-catenin^CA^ abrogated the Wee1 protein upregulation in dense monolayers, indicating that force transduction by the cadherin complex is essential for the density-dependent regulation of Wee1 (Figures 5D and S3A). Density-dependent changes in Wee1 protein levels were similarly observed in Caco-2 cells, with increased Wee1 levels corresponding to reduced mitotic events at high cell density, suggesting a universality of these findings across epithelial cell types (Figures S3B–S3E).

Wee1 regulates G2/M progression through inhibitory phosphorylation of Cdk1, the gatekeeper of mitotic entry (Parker et al., 1992). To test if this inactive state of Cdk1 is induced in dense monolayers, we immunostained epithelial monolayers with an antibody selectively recognizing Cdk1 when phosphorylated on its inhibitory Y15 residue (Cdk1 pY15). This showed very low levels of Cdk1 pY15 at low monolayer density, which were strongly increased in dense monolayers (Figures 5E and 5F). Because Cdk1 pY15 levels were elevated in virtually all cells of the dense monolayer (Figures 5E and 5F), this indicates that the negative regulation of Cdk1 occurs independently of the cell-cycle phase in which cells reside. Treatment with the selective Wee1 inhibitor MK-1775 resulted in near complete disappearance of Cdk1 pY15, demonstrating that Y15 phosphorylation of Cdk1 in dense MDCK monolayers is predominantly mediated by Wee1 (Figure 5E). Importantly, the increase in Cdk1 pY15 levels at high density was abolished by expression of α-catenin^CA^ (Figures 5G and S3F). Altogether, our data indicate that Wee1 levels and concomitant inhibitory Cdk1 phosphorylation increase with monolayer density in a manner dependent on E-cadherin mechanotransduction.

Of note, we observed that Cdk1 pY15 was not only present in the nucleus and cytosol but also prominently resided at cell-cell contacts (Figure 5E). Similarly, immunostaining for total Cdk1 showed the localization of Cdk1 at cell-cell contacts (Figure S3G). To validate this junctional localization, we induced transient CRISPR-CAS9-mediated knockout of Cdk1, which abolished the immunofluorescence signal of both total and pY15 Cdk1 in the nucleus and cytosol as well as at cell-cell junctions (Figures S3H–S3J). Given that both the junctional and cytosolic pools of Cdk1 pY15 increase with cell density, potentially one or both of these pools may contribute to the density-dependent prolongation of G2 phase.

### Wee1 accumulation is required for G2 prolongation at high cell density

Having established the elevation of Wee1 levels and downstream Cdk1 Y15 phosphorylation in dense monolayers, we next investigated whether this is responsible for the G2 prolongation in these cells. To this end, we generated two Wee1 knockout (*Wee1*^–/–^) MDCK clones through CRISPR-Cas9-based gene editing using independent guide sequences (Figures 5H, 5I, and S4A), which also showed near complete loss of Cdk1 pY15 levels in dense monolayers (Figure 5I). Of note, we did not observe any increase in DNA damage in *Wee1*^–/–^ cells under our culture conditions, as determined by γH2AX immunostaining (Figure S4B). Analysis of G2 duration showed that G2 length was comparable between *Wee1*^–/–^ clones and parental cells grown at low monolayer density (Figures 5J and S4C). This indicates that Wee1 is not essential per se for the regulation of G2/M progression in MDCK cells, potentially explained by the redundant role of Myt1 kinase in MDCK cells depleted of Wee1 (Mueller et al., 1995). However, the prolongation of G2 phase at high density was completely abolished in both *Wee1*^–/–^ clones (Figures 5J and S4C). These data demonstrate that Wee1 plays a specific role in the control of the epithelial cell cycle by prolonging G2 phase when monolayer density increases.

While expression of Wee1 is strongly increased in dense monolayers, Wee1 is not completely absent at low cell density (Figures 5A–5C). As such, the complete loss of Wee1 in our knockout cells does not fully recapitulate the Wee1 status in low-density monolayers. We also generated heterozygous Wee1 knockout MDCK cells (*Wee1*^–/+^) in which only one of the two Wee1 alleles is lost (Figure S4D). At high monolayer density, the levels of Wee1 were substantially reduced in *Wee1*^–/+^ cells compared with WT cells and remained more comparable to WT cells cultured at low density (Figures S4E and S4F). This partial reduction of Wee1 was sufficient to abrogate the density-dependent G2 regulation, as both the elevated level of Cdk1 pY15 and the increase in G2 length at high density were lost in *Wee*^–/+^ cells (Figures S4G and S4H). Altogether, these data demonstrate that prolongation of G2 phase in dense monolayers depends on the concurrent accumulation of Wee1.

### Tension-induced Wee1 degradation triggers mitotic entry

**Figure 6:**
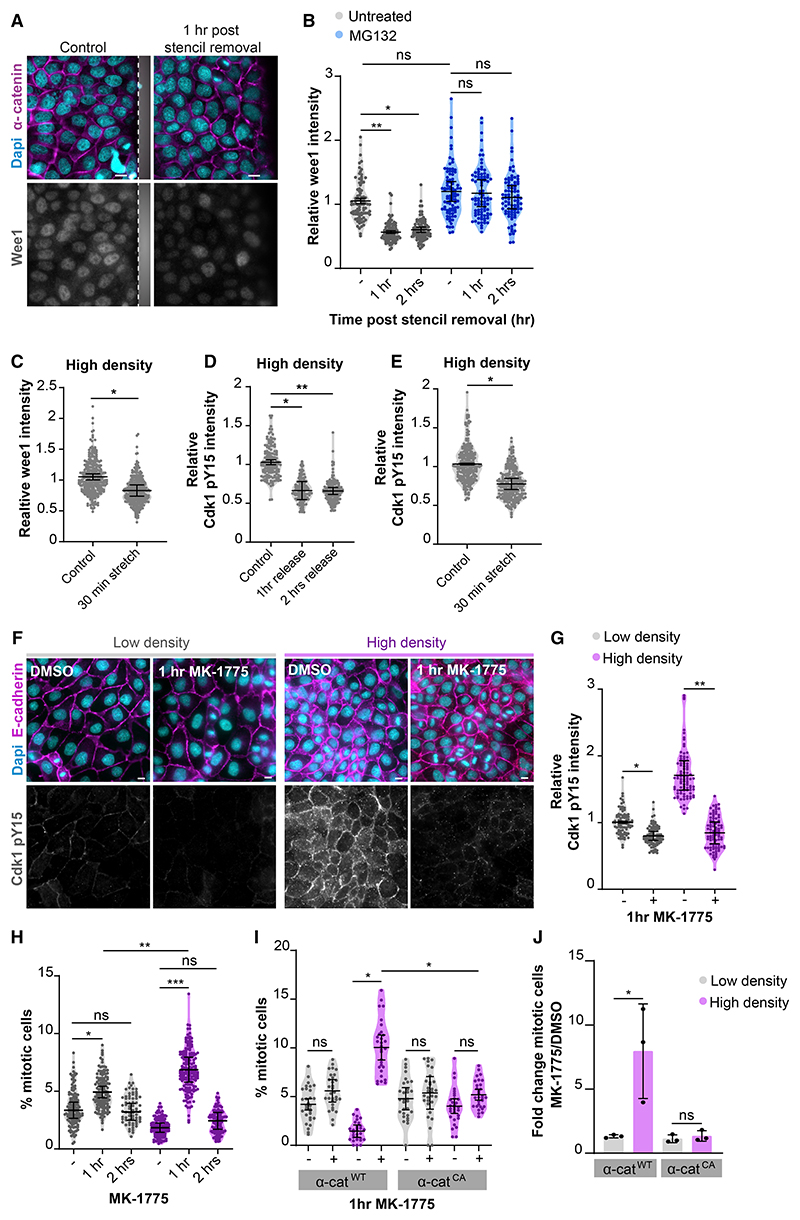
Tension-induced Wee1 degradation triggers mitotic entry (A) Immunostainings of MDCK cells for Wee1 together with α-catenin and Dapi in an unperturbed dense monolayer and 1 h after removal of a PDMS stencil (indicated in gray). (B) Quantification of Wee1 immunofluorescence intensity per cell (normalized to the level of α-catenin intensity) in unperturbed dense MDCK monolayers and 1 or 2 h following removal of the PDMS stencil, either in the absence (gray) or presence (blue) of the proteasome inhibitor MG132 (5 μM). MG132 was added 30 min before stencil removal. n = 90 cells per condition. Data were pooled from 3 independent experiments. Black bars represent the mean and SD of the individual experiments. *p = 0.012; **p = 0.0013; ns, not significant; ratio paired t test. (C) Quantification of Wee1 immunofluorescence intensity per cell (normalized to the level of α-catenin intensity) in HD MDCK monolayers with and without application of 18% uniaxial stretch (30 min). n = 290 cells per condition. Data were pooled from 4 independent experiments. Black bars represent the mean and SD of the individual experiments. *p < 0.027; ratio paired t test. (D) Quantification of Cdk1 pY15 immunofluorescence intensity per cell (normalized to the level of α-catenin intensity) in unperturbed HD MDCK monolayers and 1 or 2 h post stencil removal. n = 150 cells per condition. Data were pooled from 3 independent experiments. Black bars represent the mean and SD of the individual experiments. *p = 0.034; **p = 0.0049; ratio paired t test. (E) Quantification of Cdk1 pY15 immunofluorescence intensity per cell (normalized to the level of α-catenin intensity) in HD MDCK monolayers with and without application of 18% uniaxial stretch (30 min). n = 240 cells per condition. Data were pooled from 3 independent experiments. Black bars represent the mean and SD of the individual experiments. *p = 0.0397; ratio paired t test. (F) Immunostainings for Cdk1 pY15, together with α-catenin and Dapi, of MDCK monolayers grown at LD (left) and HD (right), either in the absence or presence of the Wee1 inhibitor MK-1775 (1 h; 500 nM). (G) Quantification of Cdk1 pY15 immunofluorescence intensity per cell (normalized to the level of E-cadherin intensity) in MDCK monolayers grown at LD (gray) or HD (magenta), either in the absence or presence of MK-1775 (1 h; 500 nM). n = 90 cells per condition. Data were pooled from 3 independent experiments. Black bars represent the mean and SD of the individual experiments. *p = 0.032; **p = 0.0093; ratio paired t test. (H) Quantification of the percentage of mitotic cells in parental MDCK monolayers grown at low (gray) and high (magenta) density, treated with DMSO or MK-1775 (500 nM; 1 or 2 h). n > 69 fields of view per condition. Data were pooled from 4 independent experiments (3 independent experiments for the 2 h time point). Black bars represent the mean and SD of the individual experiments. *p = 0.021; **p = 0.007; ***p = 0.0009, ns = not significant; paired t test. (I) Quantification of the percentage of mitotic cells in MDCK cells expressing WT or CA α-catenin-mCherry, at LD (gray) and HD (magenta), and with or without MK-1775 treatment (500 nM; 1 h). n = 30 monolayer regions per condition. Data were pooled from 3 independent experiments. Black bars represent the mean and SD of the individual experiments. *p = 0.0161 (HD WT untreated versus HD WT MK-1775 treated); *p = 0.0136 (HD WT MK-1775 treated versus HD CA MK-1775 treated), ns, not significant; paired t test. (J) Bar graph showing the fold change (mean of each individual experiment) in the percentage of mitotic cells following 1 h MK-1775 treatment (500 nM) in MDCK monolayers expressing either WT or CA α-catenin-mCherry and grown at low (gray) and high (magenta) monolayer density (from data shown in Figure 5I). The black bars represent the SD. *p = 0.022; ns, not significant; ratio paired t test. All scale bars represent 10 μm. See also Figures S5 and S6.

As we demonstrated that the rise of Wee1 levels is responsible for the increased G2 length at high cell density, we hypothesized that the induction of mitosis following elevation of intercellular forces requires downregulation of Wee1. To test this, we analyzed Wee1 levels in dense monolayers during epithelial expansion upon removal of the PDMS stencil (as in Figure 1C). Within 1 h following stencil removal, we observed a ~2-fold decrease in Wee1 levels (Figures 6A and 6B). This rapid loss of Wee1 depends on its proteasomal degradation, as in the presence of the proteasome inhibitor MG132, Wee1 levels remained unaltered following stencil removal (Figures 6B and S5A). Increasing tension in dense monolayers by application of mechanical stretch or Calyculin A treatment resulted in a similar reduction of Wee1 levels (Figures 6C and S5B–S5D), indicating that elevated mechanical tension triggers Wee1 degradation. In line with Wee1 being reduced, the levels of Cdk1 pY15 also declined within 1 h after stencil removal (Figures 6D and S5E) as well as upon application of mechanical stretch (Figures 6E and S5F) or treatment with Calyculin A (Figures S5G and S5H). Altogether, our data demonstrate that increasing mechanical tension triggers Wee1 degradation, thereby relieving the inhibitory state of Cdk1.

Finally, we tested if the downregulation of Wee1 and accompanying loss of Cdk1 pY15 are responsible for the induction of mitosis downstream of increased mechanical tension in dense monolayers. To address this, we reduced Wee1 activity with its selective inhibitor MK-1775. In dense monolayers, we observed a substantial reduction in Cdk1 pY15 levels and a 3.7-fold increase in the percentage of mitotic events upon 1 h of Wee1 inhibition (Figures 6F–6H). This burst in mitosis was transient, as the level of mitotic events was almost completely restored to basal after 2 h (Figure 6H). In contrast to dense monolayers, low-density monolayers only showed a minor, albeit significant, increase in mitosis (1.47-fold) after 1 h of Wee1 inhibition. Moreover, the total percentage of mitotic cells following Wee1 inhibition remained significantly lower in low-density monolayers compared with dense monolayers (Figure 6H). Similarly, in dense monolayers of cells expressing α-catenin^CA^, which lack a population of cells with G2 prolongation (Figure 4B), Wee1 inhibition was much less effective in increasing mitotic events than in dense α-catenin^WT^ monolayers (Figures 6I and 6J). These data indicate that specific reduction of Wee1 activity, which occurs upon elevation of tension on E-cadherin adhesions, is sufficient to relieve the density-dependent G2 prolongation and triggers rapid mitotic entry. Of note, Wee1 levels were not downregulated by direct activation of the stretch-sensitive calcium channel Piezo1, which was recently shown to be involved in the stretch-induced regulation of mitosis (Gudipaty et al., 2017) (Figure S6). Taken together, our data show that Wee1 protein levels are controlled by intercellular forces transduced by E-cadherin adhesions to establish cell density-dependent regulation of G2/M progression.

## Discussion

Our work uncovered that, in addition to conventional contact inhibition of proliferation that halts cells in G0/G1, epithelial cells possess a mechanical checkpoint that serves to coordinate G2/M progression with local variations in cell density. Mechanistically, we show this is established through mechanosensing by E-cadherin adhesions and downstream regulation of the Cdk1-inhibitory kinase Wee1. As cell density increases, tension on E-cadherin adhesions is reduced, which results in accumulation of Wee1 to maintain an inactive state of Cdk1. The density-dependent regulation of Wee1 occurs throughout the cell cycle and, once cells enter G2, is sufficient to temporarily halt cells in this cell-cycle phase. This pool of G2-halted cells is readily triggered to enter mitosis by elevation of intercellular tension, for instance during epithelial expansion upon wounding, which triggers degradation of Wee1.

While it has long been thought that cells become irresponsive to extracellular cues following G1/S progression (Pardee, 1989), several studies in different model organisms support our findings indicating G2 phase as a key point of cell-cycle control by extrinsic cues. As early as in the 1960s and 1970s, labeling studies performed in mouse and rat epithelia suggest the presence of a pool of cells that is blocked in G2 phase indefinitely, yet these cells maintain the capacity to divide in response to external stimuli, such as wounding or hormones (Pederson and Gelfant, 1970; Gelfant, 1977). Recent studies in *Drosophila, Xenopus laevis*, and *C. elegans* tissues uncovered that populations of adult stem cells are halted in G2 phase and can be triggered to divide by growth factor and nutrient signaling (Zielke et al., 2014; Otsuki and Brand, 2018; Seidel and Kimble, 2015; Morris and Spradling, 2011; McKeown and Cline, 2019). Extrinsic control of G2/M progression may thus be a widely conserved element of the eukaryotic cell cycle. Our work, supported by recent findings of stretch-induced upregulation of mitotic events (Nestor-Bergmann et al., 2019; Gudipaty et al., 2017), shows that this involves a key role for intercellular communication through mechanical forces. Moreover, our identification of density-dependent forces as a major determinant of G2 length is in line with recent work demonstrating levels of intercellular tension as a predictor of cell-cycle duration, including the combined length of S-G2-M phases (Uroz et al., 2018). The mechanical G2 checkpoint may serve to ensure precise coordination of cell division with variations in local cell density and provide a mechanism to maintain epithelial integrity by rapidly increasing cell number following cell loss without a time delay needed for progression through the entire cell cycle. Halting cells in G2 rather than cell-cycle exit may have additional advantages, as it allows for high-fidelity homologous recombination-mediated repair in response to DNA damage, thereby preserving the integrity of the genome.

Besides implications for epithelial homeostasis, the mechanical G2 checkpoint might also be involved in the control of cell division in tissue development. During embryogenesis, cells are temporarily blocked in G2 in order to coordinate cell divisions with tissue growth, differentiation, and morphogenetic movements (Bouldin and Kimelman, 2014; Ogura et al., 2011; Mata et al., 2000; Grosshans and Wieschaus, 2000; Seher and Leptin, 2000; Murakami et al., 2004; Leise and Mueller, 2002). As morphogenesis is inherently a mechanical process, variations in intercellular tension that occur during morphogenetic movements could potentially contribute to this cell-cycle regulation.

Our data identify Wee1 as a key component of the mechanical G2 checkpoint downstream of E-cadherin mechanosensing and the force-induced conformational opening of α-catenin (Figures 5 and 6). The regulation of cell-cycle entry of contact-inhibited cells by intercellular forces is similarly established by force-induced opening of α-catenin through consequent binding to Ajuba/Zyxin family proteins (Alégot et al., 2019; Dutta et al., 2018; Ibar et al., 2018; Rauskolb et al., 2014). Whether conformational opening of α-catenin directly establishes Wee1 regulation, or this involves indirect signaling (e.g., through modulation of the actin cytoskeleton), remains an important question for future investigation. Our data indicate that intercellular forces regulate Wee1 at the level of its proteasomal degradation (Figures 6B and S5A), which is known to be controlled by the ubiquitin ligases β-trcp and trigger of mitotic entry-1 (Tome-1) (Smith et al., 2007). Tome-1 has previously been identified as a potential interactor of the E-cadherin complex (Guo et al., 2014), suggesting a putative link between E-cadherin adhesions and Wee1 degradation. Intriguingly, in fission yeast, Wee1 regulation couples the timing of mitotic entry to cell growth, inducing division only once cells have achieved their appropriate size (Allard et al., 2017). This suggests an evolutionary conserved role for Wee1 in sensing physical cues and coordinating cell divisions with cell/tissue size.

Although our data demonstrate a role for E-cadherin and Wee1 in mediating the density-dependent G2/M progression, importantly, this does not exclude the contribution of other mechanotransduction mechanisms. As such, it was recently shown that induction of mitotic entry following mechanical stretch requires activation of the calcium-channel Piezo1 (Gudipaty et al., 2017). This function of Piezo has been linked to regulation of cyclin B transcription (Gudipaty et al., 2017), and we find that direct activation of Piezo1 and downstream calcium influx does not downregulate Wee1 levels in MDCK cells (Figure S6). This suggests that E-cadherin and Piezo1 may act in parallel to control force-dependent G2/M progression. Future studies may shed light on the interplay between different mechanosensing complexes in the regulation of G2/M progression in epithelial cells.

We demonstrate that G2 prolongation at high density specifically depends on accumulation of Wee1 (Figures 5J, S4C, and S4H) and, moreover, that inhibition of Wee1 is sufficient to drive these G2-halted cells into mitosis (Figures 6H–6J). Nonetheless, as the inhibitory phosphorylation of Cdk1 by Wee1 is counter-acted by Cdc25 phosphatases, the activation of Cdk1-cyclin B ultimately depends on the balance between Wee1 and Cdc25 activity (Lindqvist et al., 2009). It will therefore be interesting to explore if epithelial wounding and fluctuations in cell density specifically control Wee1 or also impact Cdc25.

We observed that Cdk1 and its pY15-phosphorylated state are not only present in the nucleus and cytosol but also reside at cell-cell contacts in epithelial cells (Figures 5E and S3G). This is in line with recent findings in *Xenopus* embryos, where a large fraction of Cdk1 and the pY15-phosphorylated pool was shown to localize to cell-cell junctions (Sandquist et al., 2018). The presence of Cdk1 pY15 at cell-cell junctions was abolished following Wee1 inhibition (Figure 5E), implying that the cytosolic Wee1 pool is able to phosphorylate Cdk1 at this site. Our data indicate that both the junctional and cytosolic Cdk1 pools undergo density-dependent regulation of Y15 phosphorylation by Wee1 (Figure 5E), yet it remains to be determined which fraction of Cdk1 is essential for the ensuing regulation of G2/M progression. It has been shown that Cdk1-cyclin B is first activated in the cytoplasm prior to mitotic entry and then stimulates its own transport to the nucleus by regulating the nuclear transport machinery (Gavet and Pines, 2010). Whether junctional Cdk1 can translocate to the nucleus, or whether it influences cytosolic Cdk1 through one of the described positive feedback mechanisms by which Cdk1 promotes its own activity (Lindqvist et al., 2009), remains to be determined. Cdk1 may also perform specific functions at cell-cell junctions. For instance, in *Xenopus* embryos, activation of the junctional Cdk1 pool during meta-phase is implicated in coupling mitotic spindle dynamics to anaphase onset (Sandquist et al., 2018). One alluring hypothesis is that regulation of the junctional Cdk1 pool may modulate cell-cell adhesion complexes throughout the cell cycle, for instance to allow their remodeling before mitotic entry to enable mitotic rounding, analogous to the regulation of focal adhesion dynamics by Cdk1 (Jones et al., 2018).

### Limitations of the study

Our findings demonstrate that the level of forces on E-cadherin adhesions and conformational regulation of α-catenin establish cell-density-dependent control of Wee1 levels and, consequently, G2 progression. Further studies are required to elucidate the connection between E-cadherin adhesions and Wee1 regulation, which will also address whether the force-induced conformational opening of α-catenin directly establishes Wee1 regulation or whether this involves an indirect cellular response, for instance through effects on the actin cytoskeleton. Although our data demonstrate an essential role for Wee1 in the force-dependent Y15 phosphorylation of Cdk1, it remains to be determined if other regulators of this phosphorylation (CDC25 phosphatases and Myt1 kinase) are similarly modulated by mechanical signals. Finally, this work is predominantly based on experimental findings in MDCK cells, and it will be important to investigate the contribution of the identified mechanical G2 checkpoint to the regulation of cell division in native epithelia using other model systems.

## Star⋆Methods

Detailed methods are provided in the online version of this paper and include the following:

- KEY RESOURCES TABLE
- RESOURCE AVAILABILITY
  ○ Lead contact
  ○ Materials availability
  ○ Data and code availability
- EXPERIMENTAL MODEL AND SUBJECT DETAILS
  - Cell lines
- METHOD DETAILS
  ○ Antibody and reagent dilutions
  ○ Generation of plasmids
  ○ Live cell microscopy and analyses
  ○ Immunofluorescence stainings and analyses
  ○ Epithelial expansion upon stencil removal and stretch assays
  ○ PIV analysis
  ○ Monolayer stress microscopy imaging and analysis
  ○ E-cadherin TsMod FRET imaging and analysis
- QUANTIFICATION AND STATISTICAL ANALYSIS

## Star ⋆ Methods

### Key Resources Table

**Table:** 

REAGENT or RESOURCE	SOURCE	IDENTIFIER
Antibodies		
α-tubulin, clone DM1A (mouse)	Sigma-Aldrich	Cat# T6199; RRID: AB_477583
Ki67 (Rabbit)	Abcam	Cat# ab15580; RRID: AB_443209
E-cadherin, DECMA-1 (Rat)	GeneTex	Cat# GTX11512, RRID: AB_381324
αE-catenin, clone 15D9 (Mouse)	Enzo	Cat# ALX-804-101-C100, RRID: AB_2050688
α-catenin (Rabbit)	Sigma-Aldrich	Cat# C2081, RRID: AB_476830
Phospho-Histone H3 Ser10, clone 3H10 (Mouse)	Millipore	Cat# 05-806, RRID: AB_310016
Wee1, clone D10D2 (Rabbit)	Cell Signaling Technology	Cat# 13084, RRID: AB_2713924
α-actinin, clone BM-75.2 (Mouse)	Sigma-Aldrich	Cat# A5044, RRID: AB_476737
Phospho-Cdk1 Tyr15 (Rabbit)	Cell Signaling Technology	Cat# 9111, RRID: AB_331460
Vinculin, clone hVin-1 (Mouse)	Sigma-Aldrich	Cat# V9131, RRID: AB_477629
Cdk1, PSTAIR (Rabbit)	Millipore	Cat# 06-923, RRID: AB_310302
Cdk1, clone POH1 (Mouse)	Cell Signaling Technology	Cat# 9116, RRID: AB_2074795
p130CAS (Mouse)	BD Biosciences	Cat# 610271, RRID: AB_397666
Phospho-Histone H2A.X Ser139, clone JBW301 (Mouse)	Millipore	Cat# 05-636, RRID: AB_309864
Chemicals, peptides, and recombinant proteins		
MK-1775	Axon Medchem	Cat# 1494; CAS 955365-80-7
Calyculin A	Enzo	Cat# BML-EI192-0100; CAS 101932-71-2
MG-132	Santa Cruz	Cat# sc-201270A; CAS 133407-82-6
Thapsigargin	Sigma-Aldrich	Cat# T9033; CAS 67526-95-8
Yoda	Tocris	Cat# 5586; CAS 448947-81-7
Rat tail Collagen I	Corning	Cat# 354236
Experimental models: Cell lines		
MDCK GII	Mays et al., 1995	RRID: CVCL_0422
MDCKGII FUCCI4	This work	N/A
MDCK GII α-catenin^WT^	This work	N/A
MDCKGII α-catenin^CA^	This work	N/A
MDCK GII α-catenin^WT^ - mTurquoise2-SLBP - H1-mMaroon1	This work	N/A
MDCK GII α-catenin^WT^ - mTurquoise2-SLBP - H1-mMaroon1	This work	N/A
MDCK GII Wee1^-/-^	This work	N/A
MDCK GII Wee1^-/+^	This work	N/A
MDCK GII Wee1^-/-^- mTurquoise2-SLBP - H1-mMaroon1	This work	N/A
MDCK GII Wee1^-/+^- mTurquoise2-SLBP - H1-mMaroon1	This work	N/A
MDCK GII GCaMP3	This work	N/A
MDCK GII E-cadherin TsMOD	Borghi et al., 2012	N/A
MDCK GII E-cadherin TsMOD ΔCyto	Borghi et al., 2012	N/A
Caco-2	ATCC	HTB-37; RRID: CVCL_0025
Oligonucleotides		
Wee1 knockout guide RNA #1: 5’-CAC CGCGCGATGAGCTTCCTGAGC-3’	This work	N/A
Wee1 knockout guide RNA #2: 50-CAC CGAAGAGCCGCAGCTT GCGGA-3’	This work	N/A
Cdk1 knockout guide RNA #1: 50-AAAC ATGGAGTTGTGTATAAGGGTC-3’	This work	N/A
Cdk1 knockout guide RNA #2: 5’-CAC CGTTGTCTATTTCAGGTACCTA-3’	This work	N/A
Recombinant DNA		
pLL3.7m-Clover-Geminin(1-110)-IRES- mKO2-Cdt(30–120) (FUCCI4)	Bajar et al., 2016	Addgene, Cat# 83841
pLL3.7m-mTurquoise2-SLBP(18–126)- IRES-H1-mMaroon1 (FUCCI4)	Bajar et al., 2016	Addgene, Cat# 83842
pInducer20 mCherry-αE-catenin^WT^	This work	N/A
pInducer20 mCherry-αE-catenin (M319G, R326E)^CA^	This work and ref. (Matsuzawa et al., 2018)	N/A
Tol2-hEF1α-H2B TagRFP-2A-GCaMP3	Kind gift from Bas Ponsioen, University Medical Center Utrecht, the Netherlands and ref. (Tian et al., 2009)	N/A
Software and algorithms		
ImageJ	National Institutes of Health and ref. (Schindelin et al., 2015)	https://imagej.net/software/fiji/
Silhouette studio	Silhouette	https://www.silhouetteamerica.com/software
GraphPad Prism-v9.1.0	GraphPad Software, LLC	https://www.graphpad.com/
Python 3.0	Python	https://www.python.org/
MATLAB	MathWorks	https://www.mathworks.com/products/matlab.html
PIVLab	Thielicke and Stamhuis, 2014	pivlab.blogspot.com
PixFRET	Feige et al., 2005	http://www.unil.ch/cig/page16989.html
Other		
PDMS sheets (thickness: 250 μm)	Stockwell Elastomerics	Cat# HT-6240
Sylgard 182 silicone elastomer kit	Dow Corning	N/A
Basic plasma cleaner	Harrick Plasma	PDC-32G
computer-controlled razor writer (Cameo, Silhouette)	Silhouette	N/A
FluoSpheres™ Carboxylate-Modified Microspheres	Invitrogen	Cat#F8810
DOWSIL™ CY 52–276 A&B	Dow Corning	N/A

### Resource Availability

#### Lead contact

Further information and requests for resources and reagents should be directed to and will be fulfilled by the lead contact, Martijn Gloerich (m.gloerich@umcutrecht.nl).

#### Materials availability

Cell lines and plasmids generated in this study are available from the lead contact without restriction.

### Experimental Model and Subject Details

#### Cell lines

Madin-Darby canine kidney (MDCK) GII cells were cultured at 37°C and 5% CO_2_ in low glucose DMEM containing 10% FBS, 1 g/L sodium bicarbonate, and penicillin/streptomycin. Live-cell imaging was performed with the same media formulations. Human colorectal adenocarcinoma Caco-2 cells were cultured in high glucose DMEM supplemented with 1% Non-Essential Amino Acid Solution (Sigma) and containing 10% FBS and penicillin/streptomycin. MDCK E-cadherin TSMod and TSModΔCyto cell lines are previously described (Borghi et al., 2012), all other cell lines were generated using transfection reagent Lipofectamine 3000 (Thermo Fisher Scientific) or by lentiviral transductions and sorted by FACS to obtain monoclonal lines. Wee1 knockout lines were generated by expression of a SpCas9-GFP plasmid containing a Wee1-specific gRNA (guide 1: 5′-CACCGCGCGATGAGCTTCCTGAGC-3′, guide 2: 5′-CACCGAAGAGCCGCAGCTTGCGGA-3′). Knockout lines were verified by immunofluorescence and Western blot analysis for endogenous Wee1. Cdk1 knockout cells were generated by transient expression of a SpCas9-GFP plasmid containing a Cdk1-specific gRNA (guide 1: 5′-AAACATGGAGTTGTGTATAAGGGTC-3′, guide 2: 5′-CACCGTTGTCTATTTCAGGTACCTA-3′), and verified by Cdk1 immunostainings. For cells with doxycycline-induced ectopic expression of α-catenin, cells were cultured in medium containing 2 μg/mL doxycycline for at least 24 h. All cell lines were regularly tested for the absence of mycoplasma.

### Method Details

#### Antibody and reagent dilutions

The following commercial antibodies were used at the indicated concentrations for Western blot (WB) and immunofluorescence (IF): α-tubulin (DM1A, Sigma-Aldrich; Cat#T6199, 1:1000 IF), Ki67 (Abcam; Cat#ab15580; 1:1000 IF), E-cadherin (DECMA-1, GeneTex; Cat#GTX11512, 1:1000 IF), αE-catenin (Enzo; Cat#ALX-804-101-C100, 1:500 IF), α-catenin (Sigma-Aldrich; Cat#C2081, 1:500 IF), Phospho-Histone H3 Ser10 (3H10, Millipore; Cat#05–806, 1:1000 IF), Wee1 (D10D2, Cell signaling; Cat#13084, 1:500 IF, 1:1000 WB), α-actinin (BM-75.2, Sigma-Aldrich; Cat#A5044, 1:500 IF), phospho-Cdk1 Tyr15 (Cell signaling; Cat#9111, 1:250 IF), Vinculin (hVIN-1, Sigma-Aldrich; Cat#V9131, 1:250 IF), Cdk1 (PSTAIR; Millipore; Cat#06–923; 1:500), Cdk1 (POH1, Cell signaling; Cat#9116, 1:250 IF), p130CAS (BD Biosciences; Cat#610271, 1:3000 WB), phospho-Histone H2A.X Ser139 (JBW301; Millipore; Cat#05–636, 1:1000 IF).

The following reagents were used at the indicated concentrations: MK-1775 (Axon Medchem; Cat#1494, 500 nM), Calyculin A (Enzo; Cat#BML-EI192-0100, 10 ng/mL), MG132 (Santa Cruz; Cat#sc-201270, 5µM), Thapsigargin (Sigma-Aldrich; Cat#T9033, 1 µM), Yoda (Tocris; Cat#5586, 10 µM).

#### Generation of plasmids

The following plasmids are described elsewhere: pLL3.7m-Clover-Geminin(1–110)-IRES-mKO2-Cdt(30–120) and pLL3.7m-mTur-quoise2-SLBP(18–126)-IRES-H1-mMaroon1 (FUCCI4; (Bajar et al., 2016), Tol2-hEF1α-H2B TagRFP-2A-GCaMP3 (Tian et al., 2009) (kind gift from Bas Ponsioen, University Medical Center Utrecht, the Netherlands). The pInducer20 mCherry-αE-catenin plasmid was generated using In-Fusion cloning (inserting mCherry-αE-catenin [GI : 49935; (Oldenburg et al., 2015)] in a pInducer20 vector [Addgene; #44012]). Finally, the M319G, R326E αE-catenin (α-catenin^CA^; ref. (Matsuzawa et al., 2018)) mutant was created using PCR mutagenesis.

#### Live cell microscopy and analyses

For live cell imaging, cells were seeded within rectangular microwells that were cut using a computer-controlled razor writer (Cameo, Silhouette) in 250 μm thick silicone PDMS sheeting (Bisco HT-6240, Stockwell Elastomers). These silicone stencils were placed on top of a glass-bottom dish (WillCo-dish) pre-coated with Rat tail Collagen I (Corning). Cells were imaged on a Zeiss Cell Observer equipped with Orca Flash 4.0 camera (Hamamatsu) using a 40× objective (NA = 1.1) (Figure 2) or a 10× objective (NA = 0.3) (Figures 3A and 3B), or on a Nikon Spinning Disc confocal microscope using a 20× objective (NA = 0.75) (Figures 4B, 5J, S4C, S4H, and S6C–S6E). Imaging was performed in temperature- and CO_2_-controlled incubators, using Zen image acquisition software and NIS-Elements software, respectively.

For cell cycle analyses, MDCK cells expressing FUCCI4, or mTurquoise-SLBP(18–126) and Histone H1-mMaroon to monitor only G2 length, were imaged with a time interval of 10 min. The length of each cell cycle phase was determined per cell based on changes in expression levels of the different cell cycle markers using ImageJ software (National Institutes of Health). The length of G0/G1 is defined as the time from completion of cell division (and concomitant start of Cdt1 expression) to the start of Cdt1 degradation; the length of S-phase is defined as the time from the start of Cdt1 degradation (and simultaneous increase in geminin expression) to the start of SLBP degradation; the length of G2-phase is defined as the time from the start of SLBP degradation to nuclear envelope breakdown. To monitor intracellular calcium levels, MDCK cells expressing the GCaMP3 calcium sensor were imaged with a time interval of 30 s, before and after incubation with the indicated compounds. The GCaMP3 fluorescence signal (mean gray value) was measured over time in the cytosol of single cells using ImageJ software (National Institutes of Health).

#### Immunofluorescence stainings and analyses

For immunofluorescence stainings, cells within microwells in silicone stencils were fixed with 4% formaldehyde (Sigma-Aldrich), permeabilized with 0.2% Triton X-100 (Sigma-Aldrich), blocked in buffer containing 1% BSA (Sigma-Aldrich), 1% goat serum (Life Technologies), and 1% donkey serum (Jackson Immunoresearch), and incubated with the indicated primary and Alexa-conjugated secondary antibodies (Life technologies), together with Dapi (Sigma-Aldrich) where indicated. Cells were imaged on a Zeiss LSM880 scanning confocal microscope using a 40× objective (NA = 1.1), with the exception of Figures 1A, 1D, 4E, 6F, S1A, S1F, S2E, S3I, and S5E–S5G), which were imaged on a Zeiss Cell Observer equipped with Orca Flash 4.0 camera (Hamamatsu) using a 403 objective (NA = 1.1), using Zen image acquisition software.

The fluorescence intensities (mean gray value) of Wee1 and Cdk1 pY15 were measured in individual cells within the monolayer (with at least 10 cells analyzed per field of view) using ImageJ software (National Institutes of Health) and normalized to the intensity of co-stained control proteins as indicated. To compare results between different experiments, all values within one experiment were scaled to the median of the control condition of that experiment. All analyzed cells were imaged on a Zeiss Cell Observer equipped with Orca Flash 4.0 camera (Hamamatsu) using a 40× objective (NA = 1.1) and included for analyses in an un-biased manner. Statistical analyses were performed on the means of independent experiments of the original (not re-scaled) data.

#### Epithelial expansion upon stencil removal and stretch assays

To mimic epithelial wounding and induce epithelial expansion, approximately 24 h after seeding cells in microwells in PDMS stencils placed on top of a glass-bottom dish (WillCo-dish) pre-coated with Rat tail Collagen I (Corning), the PDMS stencil was carefully removed and cells were fixed at the indicated timepoints. Mitotic cells were visualized by immunostaining for phospho-Histone H3 Ser10.

For stretch assays, MDCK cells were plated on a PDMS silicone-based cell stretching device (previously described by (Hart et al., 2017). For this, PDMS elastomer and curing agent (Sylgard 182, Dow Corning) were mixed at 10:1 wt/wt ratio and poured over a 3D-printed mold and baked at 65°C overnight. To smoothen the surface the device was stamped on a thin layer of uncured PDMS, followed by baking at 65°C for 3 h. Subsequently, the device was bonded with the silicone membrane using plasma activation of the surface (PDC-32G, Harrick Plasma). Following coating with rat tail collagen type I (Corning), MDCK cells were seeded in the central channel. One day following plating, cells were stretched using a pneumatic valve to control vacuum pressure in the two neighboring side channels.

#### PIV analysis

Cell motility within monolayers was assessed by Particle Image Velocimetry using the MATLAB add-on PIVLab (pivlab.blogspot.com) (Thielicke and Stamhuis, 2014). First, 16-bit phase contrast movies (time resolution 10 min/frame, spatial resolution 59*10^6^ pixels/cm^2^) of MDCK cells at indicated monolayer densities were converted to 8-bit in ImageJ. Images were loaded into PIVlab in the time-resolved image sequencing style and motility vectors were calculated using single pass direct cross correlation with an interrogation window of 24 × 24 pixels and a 50% overlap. Abnormally large vectors (vector magnitude >5 pixels/frame) were excluded from analysis. Vector magnitude heat maps were created using a parula color map (range 0–7 pixels/frame). Vector magnitude plots were created with GraphPad Prism (v9.1.0).

#### Monolayer stress microscopy imaging and analysis

Soft elastomeric silicone gels of 12.6 kPa stiffness were prepared adapting a previously published protocol (Bergert et al., 2016; Latorre et al., 2018). The two polydimethylsiloxane (PDMS) components CY52-276A and CY52-276B (Dow Corning Toray) were mixed at a 9:10 ratio, spin-coated on glass-bottom dishes (35-mm, no. 0 coverslip thickness, Mattek) and cured overnight. Soft PDMS gels were subsequently treated with (3-aminopropyl)triethoxysilane (APTES, Sigma-Aldrich, cat. no. A3648) diluted at 5% in absolute ethanol, rinsed 3 times with ethanol 96% and once with milli-Q water. Samples were then incubated for 5 min with a filtered (220 nm, Millex® - GP) and sonicated solution of 200-nm-diameter red fluorescent carboxylate-modified beads (FluoSpheres, Invitrogen) in sodium tetraborate (3.8 mg/ml, Sigma-Aldrich), boric acid (5 mg/ml, Sigma-Aldrich) and 1-ethyl-3-(3-dimethylaminopropyl) carbodiimide (EDC, 0.1 mg/ml, Sigma-Aldrich). Gels were rinsed 3 times with milli-Q water and dried in the oven for 15 min at 60 °C. To prepare the PDMS gels for patterning after being coated with fluorescent beads, the surface of the gels was treated with a 1 mg/mL poly-lysine (PLL, Sigma) solution for 1h, then washed with HEPES buffer (pH = 8.4). A solution of 50 mg/mL mPEG (MW 5,000) - succinimidyl valerate (SVA) (Laysan Bio) dissolved in the same HEPES buffer was applied to passivate the surface for 1h, and after washing with Milli-Q water, micropatterns were generated by using a UV-activated mPEG-scission reaction spatially controlled by the system PRIMO (Alvéole) mounted on an inverted Nikon Eclipse Ti microscope. In the presence of a photo-initiator compound (PLPP, Alvéole), the antifouling properties of the PEGylated substrate are tuned by exposure to near-UV light (375 nm) and after illumination (800 mJ/mm^2^) through a 20× objective PLL is exposed. After rinsing with PBS, a mixture of rat tail type I collagen (8.33%, First Link (UK) Ltd.) and fluorescent Fibrinogen (647 nm, 1.67%, Invitrogen) dilluted in PBS was incubated at room temperature for 5 min in order to coat the PEG-free PLL with the protein solution, and excess protein was washed out. Next, cells were plated (400–1200 cells/mm^2^) to obtain the desired densities after 24h, and the fluorescence signals of the beads and cells were acquired with an automatic inverted microscope (Nikon Eclipse Ti), with a spinning disk confocal unit (CSU-W1, Yokogawa), Zyla sCMOS camera (Andor, image size 2,048 × 2,048 pixels) controlled with Micro-Manager (Stuurman et al., 2007, 2010), using a 40× objective (NA 0.75, air) and thermal CO_2_ and humidity control. A tile of each island with 20% overlap was imaged using a motorized stage. The 2D displacement field of the top layer of the gel was measured using a custom-made PIV software in MATLAB (MathWorks), by comparing the beads image of the deformed gel with a reference image taken after trypsinization. Traction forces were calculated using finite-thickness Fourier transform traction force microscopy (Trepat et al., 2009). Flat-field correction of the fluorescence signal of the individual tiles of the beads field was performed (Model and Burkhardt, 2001; Model, 2006). The corrected tiles were then stitched by using ImageJ’s Grid/Collection stitching plugin. Monolayer tension was calculated from the traction fields using Monolayer Stress Microscopy as described in (Tambe et al., 2011, 2013; Serrano et al., 2019), which was implemented as a custom-made software in Python 3 using NumPy, SciPy, Matplotlib, scikit-image, pandas, pyFFTW, opencv and cython.

#### E-cadherin TsMod FRET imaging and analysis

E-cadherin TsMOD FRET experiments were performed as described in (Canever et al., 2021). MDCK cells expressing either E-cadherin TsMod or the force-insensitive control sensor E-cadherin TsModΔCyto were seeded at increasing densities on glass-bottom wells coated with Collagen IV. Spectral images were acquired on an LSM 780 confocal microscope (Zeiss) with a 63×/1.4NA oil-immersion objective. mTFP1 was excited by the 458-nm line of a 30-mW argon laser. Emission was sampled at a spectral resolution of 8.7 nm within the 470- to 600-nm range. Fluorescent images were analyzed in ImageJ using the Fiji distribution (http://fiji.sc/wiki/index.php/Fiji) and the publicly available PixFRET (Feige et al., 2005) plugin [http://www.unil.ch/cig/page16989.html]. All channels were background-subtracted, Gaussian smoothed (radius = 1 pixel), and thresholded (above the first ~3–5% of the 12-bit range). The FRET index was computed as *I*_EYFP_/(*I*_mTFP_ + *I*_EYFP_) with *I* the intensity in the channel of maximum emission of the corresponding fluorophore. For quantification, the acceptor channel was used to segment the cell–cell contact regions, and index was averaged over the segmented cell–cell contacts.

### Quantification and Statistical Analysis

Immunofluorescent images were analyzed using ImageJ software (National Institutes of Health; ref. (Schindelin et al., 2015)) and statistical analyses were performed using Prism software (GraphPad). A value of p < 0.05 was considered significant. Further details regarding the quantification methods used for each experiment are provided in the method details. Details regarding statistical tests, n values and p values can be found in the *Figure Legends*.

## Supplementary Material

Supplemental information

## Data Availability

All data reported in this paper will be shared by the lead contact upon request. This paper does not report original code. Any additional information required to reanalyze the data reported in this paper is available from the lead contact upon request.

## Highlights

- E-cadherin adhesions mechanically sense cell density to control cell division rate
- Reduced tension on E-cadherin at high cell density prompts accumulation of Wee1
- Wee1-mediated inhibition of Cdk1 prolongs G2 phase in dense epithelia
- Increased tension triggers Wee1 degradation and mitotic entry of G2-halted cells

## In brief

Donker et al. show that epithelia adapt their rate of cell division to fluctuations in cell number by sensing corresponding changes in intercellular forces. These forces are transduced by E-cadherin adhesions to control cell-cycle progression through G2 phase via regulation of the kinase Wee1 and downstream inhibition of Cdk1.
