# Supplementary material for: A mechanical G2 checkpoint controls epithelial cell division through E-cadherin-mediated regulation of Wee1-Cdk1
Source: Cell Rep. Author manuscript; Available in PMC 2025 Jan 24. (PMC7617330; doi:10.1016/j.celrep.2022.111475)
Supplement: Supplemental information [file EMS202703-supplement-Supplemental_information.pdf]

Cell Reports, Volume *41*

Supplemental information

A mechanical G2 checkpoint controls epithelial cell division through E-cadherin-mediated regulation of Wee1-Cdk1

Lisa Donker, Ronja Houtekamer, Marjolein Vliem, François Sipieter, Helena Canever, Manuel Gómez-González, Miquel Bosch-Padrós, Willem-Jan Pannekoek, Xavier Trepat, Nicolas Borghi, and Martijn Gloerich

Donker et al., Figure S1

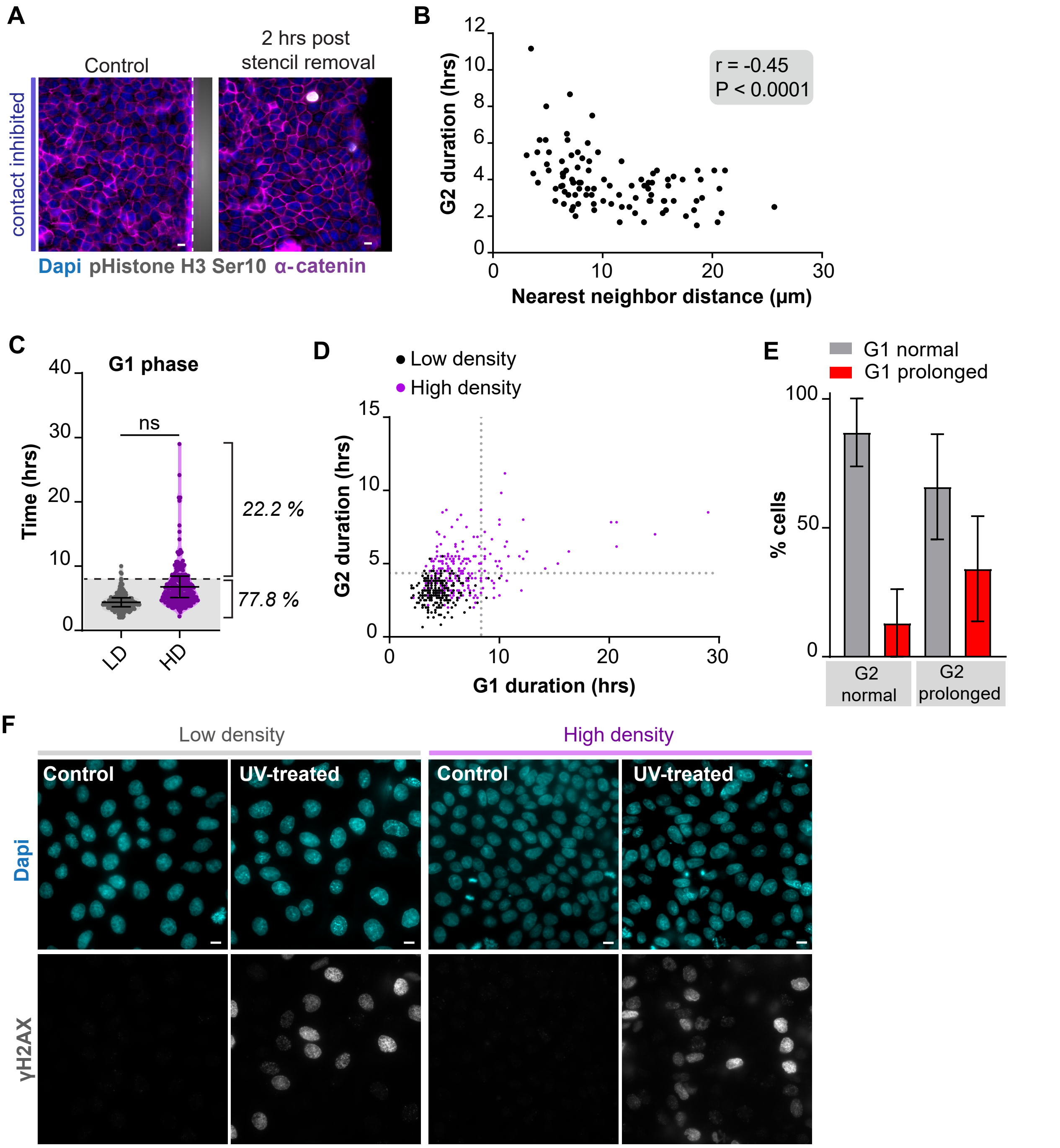

Supplementary Figure 1. Density-dependent regulation of G1- and G2 phases, Related to Figures 1 and 2.
(A) Immunostaining of MDCK monolayers grown at contact-inhibited density (CIP; see Figure 1B) without and 2 h after induction of epithelial expansion by removal of the PDMS stencil, for phospho-Histone H3 Ser10 together with α-catenin and Dapi. The PDMS stencil is indicated in grey, and the dotted line indicates the border between the stencil and the monolayers of cells.
(B) Quantification of the duration of G2 phase (h) of cells grown at various densities, correlated to the local nearest neighbor distance (NND; μm). Data shown is from one representative experiment. r = -0.45, P < 0.0001; Pearson correlation.
(C) Quantification of the duration of G1 phase (h) in monolayers grown at low (grey) and high (magenta) density (same data as in figure 2C), in which the 99% percentile of the duration of G1 in low-density monolayers is indicated (8 h, dashed line), with 22.2 ± 16.07% of cells in high-density monolayers showing a prolonged G1 duration of more than 8 h. Data were pooled from 3 independent experiments. Black bars represent the mean and SD of the individual experiments. ns = not significant; paired t-test.
(D) Correlation of G1- and G2 length within individual cells at low- (black) and high- (magenta) monolayer density. The dotted lines indicate the 99 percentiles of G1 (x-axis; 8 hrs) and G2 (y-axis; 4.5 hrs) duration of cells at low density. n = 225 cells per condition. Data were pooled from 3 independent experiments.

(E) Bar graph showing the correlation between G1 and G2 length in cells at high monolayer density. The majority of cells with a prolonged G2 phase (65.9 ± 20.4 %) did not show a prolongation of G0/G1 phase earlier in their cell cycle. Data were pooled from 3 independent experiments. Black bars represent the mean and SD of the individual experiments.
(F) Immunostaining of MDCK monolayers grown at low (left) and high (right) density, with and without UV irradiation (20 J/m^2) for the DNA damage marker γH2AX (visualizing DNA double strand breaks), together with Dapi.
All scale bars represent 10 μm.

Donker et al., Figure S2

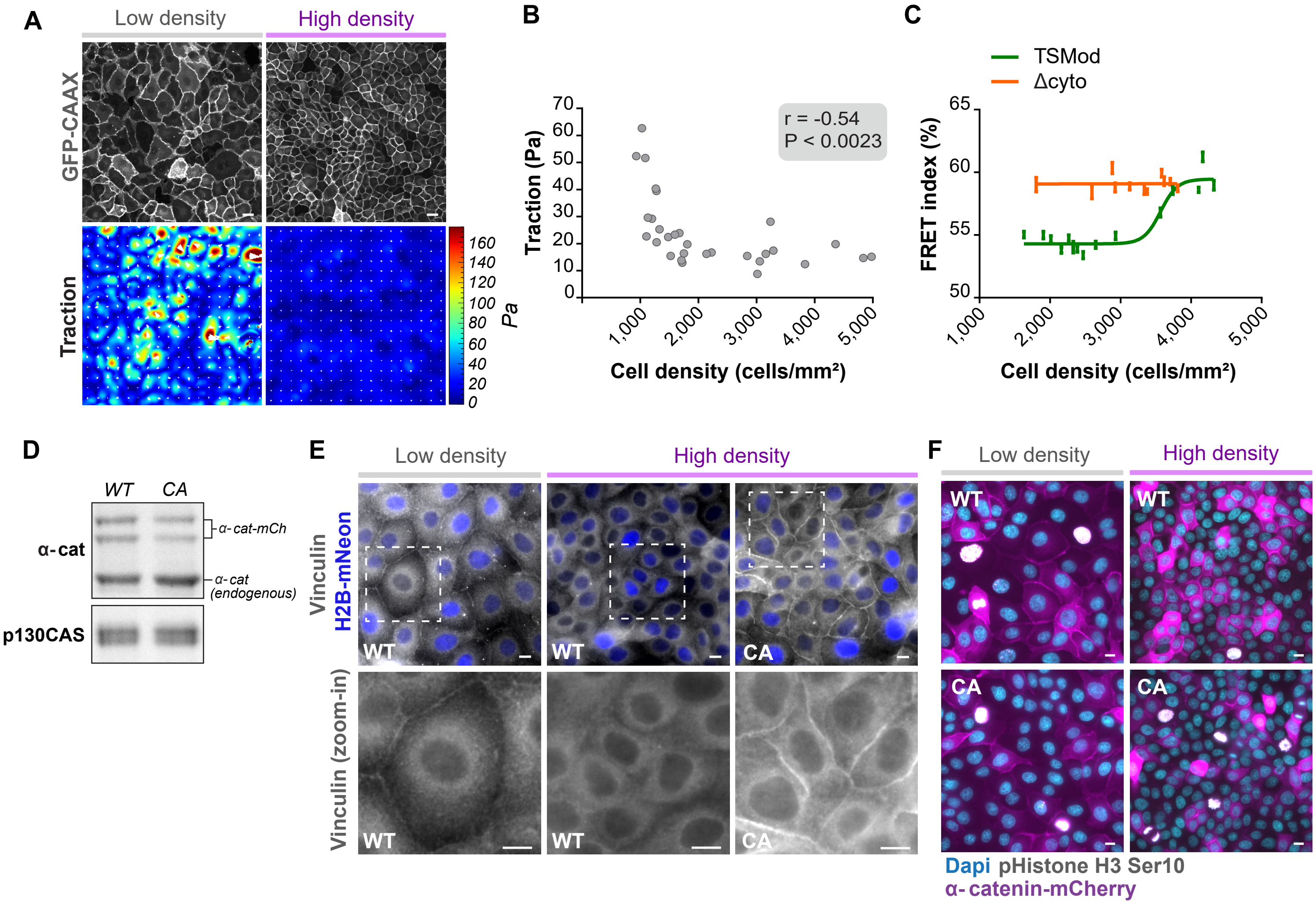

Supplementary Figure 2. Force measurements and characterization of cells expressing the conformationally active α-catenin mutant, Related to Figures 3 and 4.
(A) Representative examples of MDCK monolayers expressing GFP-CAAX, grown at low and high density with maps of traction forces (Pa). Scale bars represent 20 µm.
(B) Quantification of average traction forces at various MDCK monolayer densities. n = 30; data were pooled from 7 independent experiments. r = -0.54, P < 0.0023; Pearson correlation.
(C) Graph showing the correlation between the FRET index (%) and cell density (cells/mm²) of individual cell-cell contacts of cells expressing E-cadherin TsMod (green) or the E-cadherin ΔCyto TsMod negative control sensor (orange). Data were pooled from two independent experiments (same data as in figure 3F).
(D) Western blot of lysates from MDCK cells ectopically expressing either wildtype (WT) or constitutively open (CA) α-catenin-mCherry, probed for α-catenin and p130CAS.
(E) Immunostainings of MDCK cells expressing either wildtype (WT) or constitutively open (CA) α-catenin-mCherry for endogenous vinculin. At low monolayer density tension on E-cadherin adhesions is high (Figures 3F and 3G), causing α-catenin to unfold and recruit vinculin to cell-cell junctions. At high monolayer density, tension is low (Figures 3F and 3G) and α-catenin adopts a closed conformation and consequently, vinculin is released from cell-cell contacts. In cells expressing the α-catenin^{CA} mutant, which is constitutively in the open conformation irrespective of changes in tension, vinculin is continuously bound to α-catenin and thus enriched at cell-cell contacts, even at high density. Scale bars represent 10 µm.
(F) Immunostaining of MDCK cells expressing either wildtype (WT) or constitutively open (CA) α-catenin-mCherry (grown at low and high monolayer density) for phospho-Histone H3 Ser10 together with Dapi. Scale bars represent 10 µm.

Donker et al., Figure S3

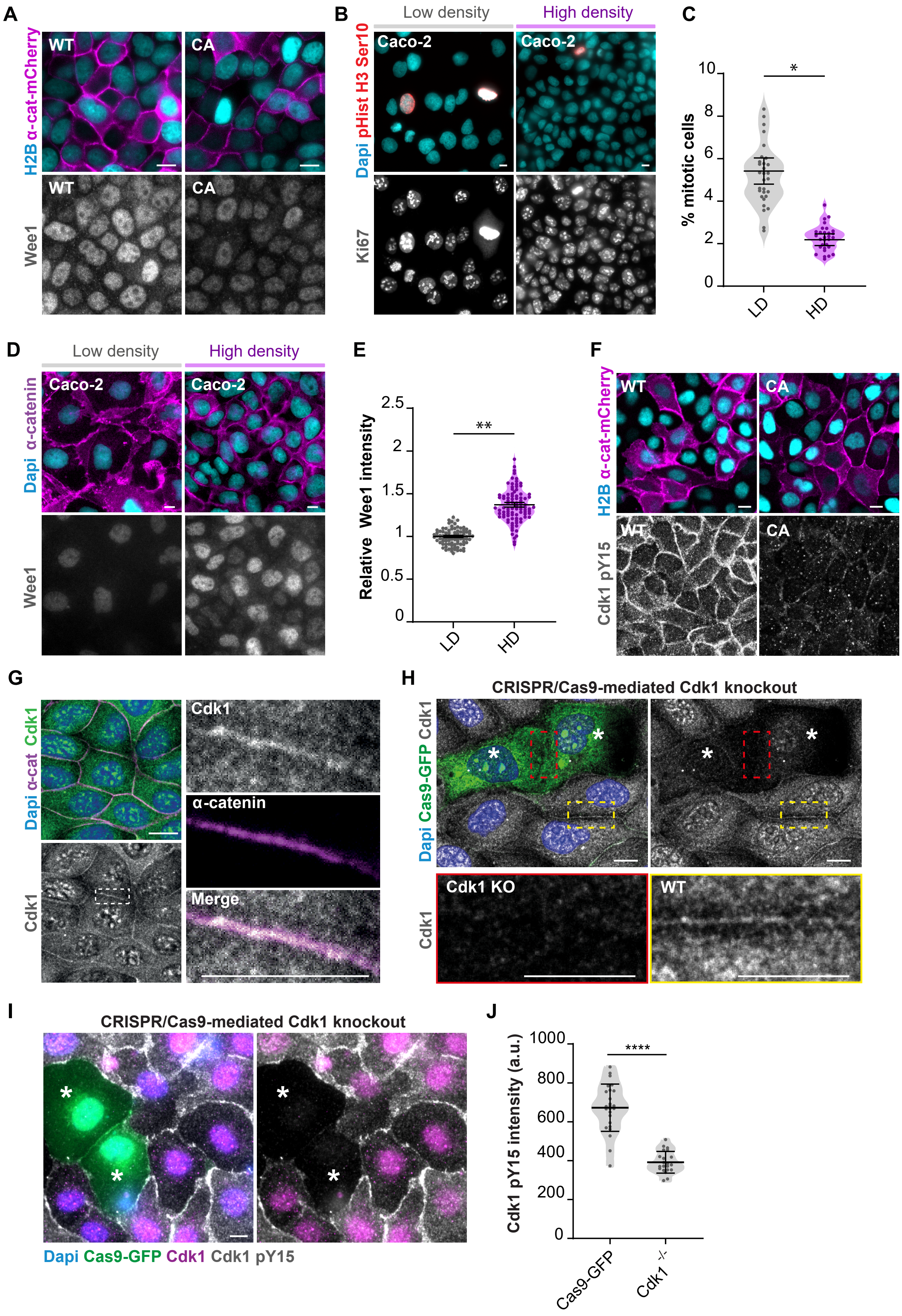

Supplementary Figure 3. Analyses of Wee1 and Cdk1 pY15 levels and validation of immunostainings, Related to Figure 5.

(A) Immunostainings of MDCK cells cultured at high density and expressing either wildtype (WT) or constitutively open (CA) α-catenin-mCherry, together with H2B-mNeon, for Wee1.
(B) Immunostainings of Caco-2 monolayers, grown at low and high density, for the proliferation marker Ki67 together with phospho-Histone H3 and Dapi to visualize mitotic cells.
(C) Quantification of the percentage of mitotic cells in Caco-2 monolayers grown at low and high density. n = 30 monolayer regions per condition. Data were pooled from 3 independent experiments. Black bars represent the mean and SD of the individual experiments. *P = 0.022; paired t-test.
(D) Caco-2 monolayers grown at low and high density and immunostained for Wee1 and α-catenin, together with Dapi.
(E) Quantification of Wee1 immunofluorescence intensity per cell (normalized to the level of α-catenin) in Caco-2 cells grown at low and high monolayer density. n = 90 cells per condition. Data were pooled from 3 independent experiments. Black bars represent the mean and SD of the individual experiments. *P = 0.0003; ratio paired t-test.
(F) Immunostainings of MDCK cells cultured at high density and expressing either wildtype (WT) or constitutively open (CA) α-catenin-mCherry, together with H2B-mNeon, for Cdk1 pY15.
(G) Immunostaining of MDCK cells for Cdk1 and α-catenin, together with Dapi.
Inset shows an individual cell-cell contact. Note that cells were fixed with methanol in order to visualize the junctional pool of Cdk1.
(H) Immunostainings of MDCK cells transiently expressing CRISPR/Cas9 with a Cdk1-targeting sequence, resulting in Cdk1 knockout, for Cdk1 together with Dapi. Note that cells were fixed with methanol in order to visualize the junctional pool of Cdk1. Cas9-GFP expressing cells were visualized by Cas9 immunostaining. Cas9-positive cells with Cdk1 knockout are indicated by white asterisks. Inset shows a cell-cell contact between two knockout cells (red), and between two wildtype cells (yellow).
(I) Immunostainings of MDCK cells transiently expressing CRISPR/Cas9 with a Cdk1-targeting sequence, resulting in Cdk1 knockout, for Cdk1 and Cdk1 pY15. Cells expressing Cas9-GFP and showing Cdk1 (pY15) depletion are indicated by white asterisks.
(J) Quantification of Cdk1 pY15 intensity in individual $Cdk1^{-/-}$ cells (expressing Cas9-GFP with Cdk1-targeting sequence, and in which total Cdk1 levels were decreased) and in cells expressing a Cas9-GFP control vector. n = 25 cells (Cas9-GFP), n = 21 cells ($Cdk1^{-/-}$). Data were derived from one representative experiment. Black bars represent the mean and SD. ****P < 0.0001; Mann-Whitney.
All scale bars represent 10 μm.

Donker et al., Figure S4

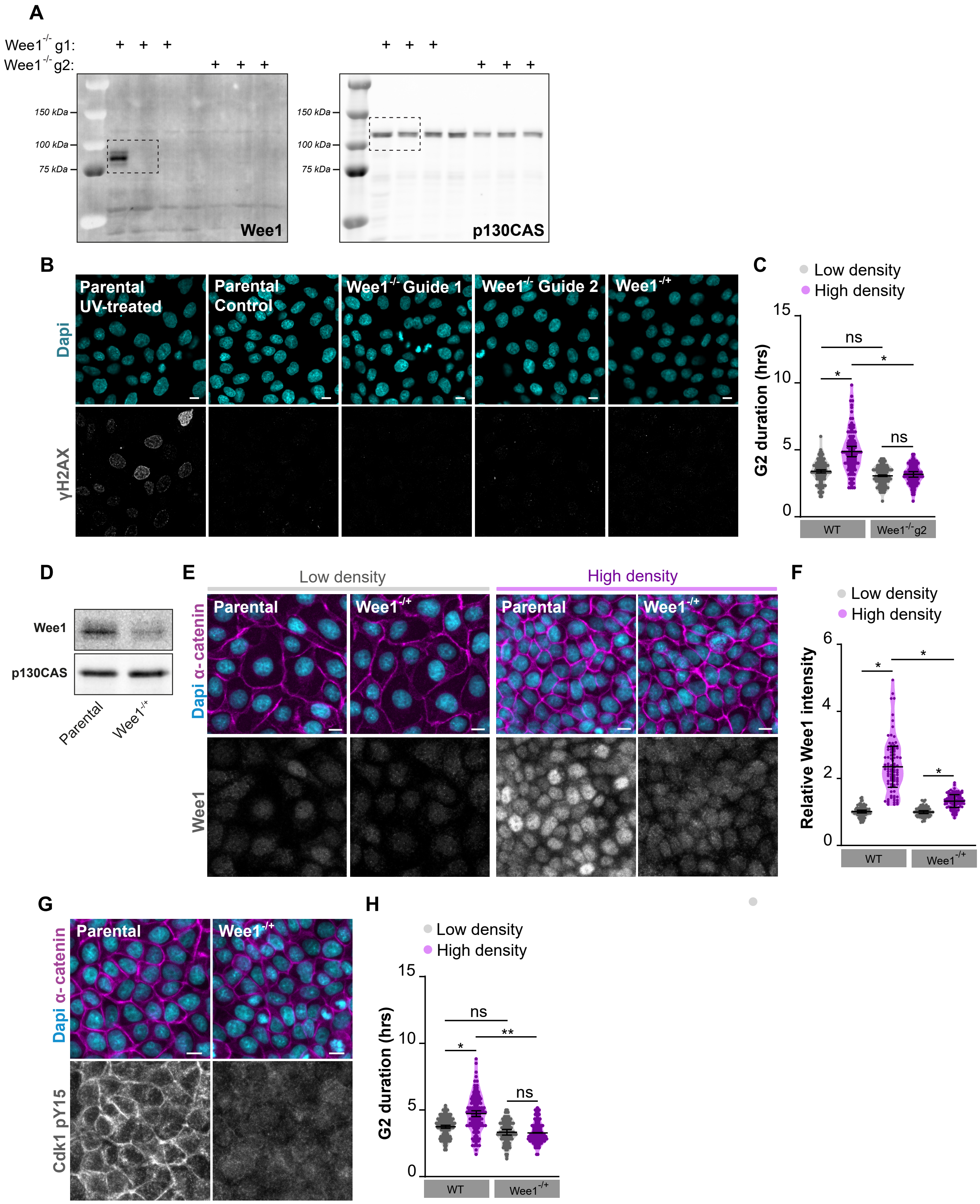

Supplementary Figure 4. Additional characterization and validation of Wee1 knockout cells, Related to Figure 5.
(A) Uncropped Western blot (same as shown in Figure 5H) of lysates from parental and 6 different *Wee1*$^{-/-}$ MDCK clones (generated with two independent guide sequences; guide 1 and guide 2) probed for Wee1 and p130CAS. The dotted line indicates the cropped region of the Western blot that is shown in Figure 5H.
(B) Immunostainings of parental MDCK cells (with and without UV irradiation; 20 J/m^2), *Wee1*$^{-/-}$ and *Wee1*$^{-/+}$ MDCK cells, for the DNA damage marker γH2AX together with Dapi.
(C) Quantification of the duration of G2 (h), based on expression of mTurquoise-SLBP(18-126), in parental and *Wee1*$^{-/-}$ (guide 2) MDCK cells grown at low and high monolayer density. n = 120 cells per condition. Data were pooled from 3 independent experiments. Black bars represent the mean and SD of the individual experiments. *P = 0.0142 (low density WT vs. high density WT), *P = 0.0168 (high density WT vs. high density *Wee1*$^{-/-}$ guide 2), ns = not significant; paired t-test.
(D) Western blot of lysates from parental and *Wee1*$^{-/+}$ MDCK cells (grown at high monolayer density) probed for Wee1 and p130CAS.
(E) Immunostainings of parental and *Wee1*$^{-/+}$ MDCK cells, grown at low (left) and high (right) monolayer density, for Wee1, together with α-catenin and Dapi.
(F) Quantification of Wee1 immunofluorescence intensity per cell (normalized to the level of α -catenin intensity) in parental and *Wee1*$^{-/+}$ MDCK cells grown at low and high monolayer density. n = 90 cells per condition. Data were pooled from 3 independent experiments. Black bars represent the mean and SD of the individual experiments. *P = 0.029 (low density WT vs. high density WT); *P = 0.046 (low density *Wee1*$^{-/+}$ vs high density *Wee1*$^{-/+}$); *P = 0.021 (high density WT vs. high density *Wee1*$^{-/+}$); ratio paired t-test.
(G) Immunostainings of parental and *Wee1*$^{-/+}$ MDCK cells cultured at high density for Cdk1 pY15, together with α-catenin and Dapi.
(H) Quantification of the duration of G2 (h), based on expression of mTurquoise-SLBP(18-126), in parental and *Wee1*$^{-/+}$ MDCK cells grown at low and high monolayer density. n = 120 cells per condition. Data were pooled from 3 independent experiments. Black bars represent the mean and SD of the individual experiments. *P = 0.022; **P = 0.0078, ns = not significant; paired t-test.
All scale bars represent 10 μm.

Donker et al., Figure S5

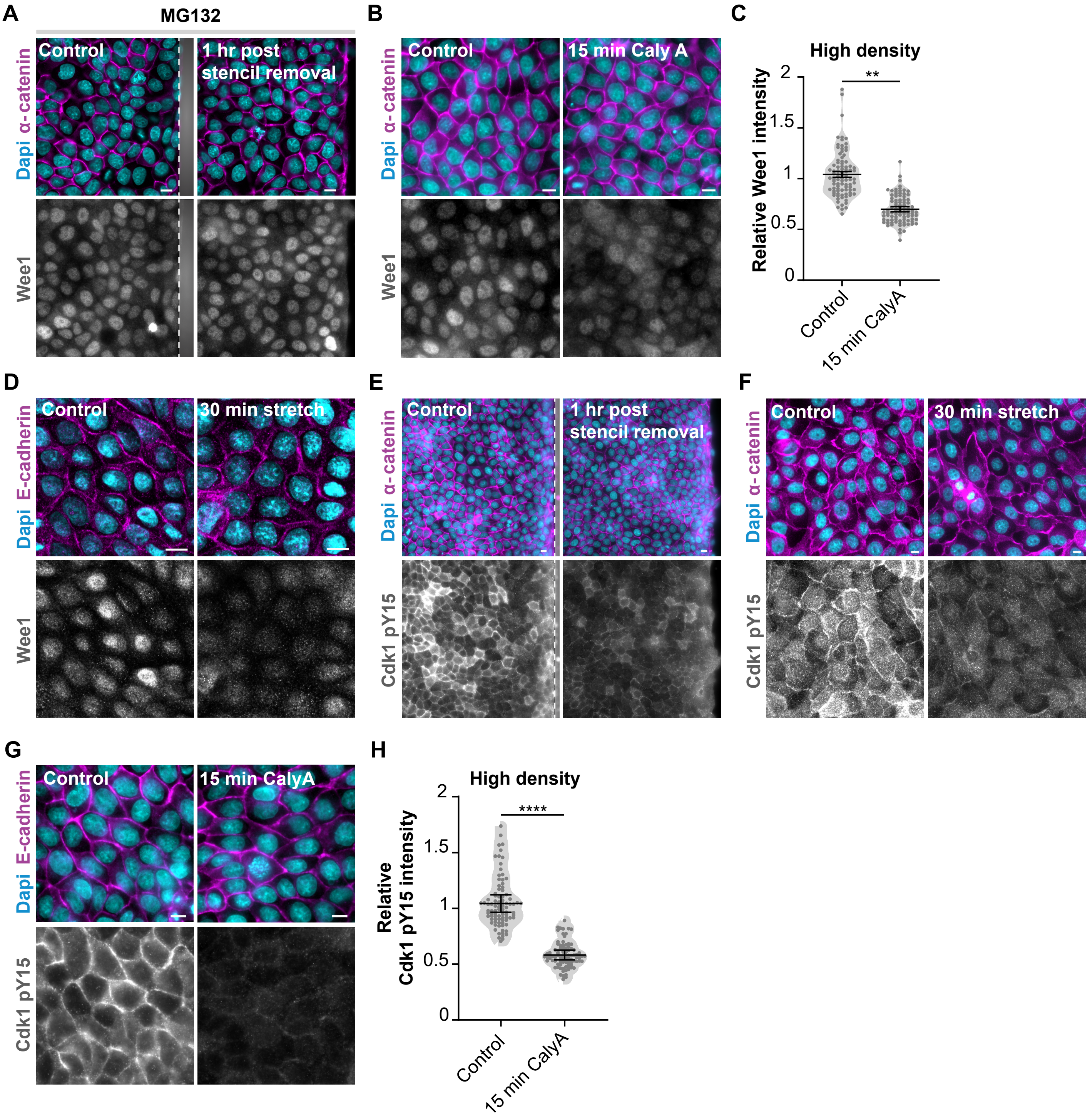

Supplementary Figure 5. Reduction of Wee1 and Cdk1 pY15 levels following elevation of intercellular forces, Related to Figure 6.

(A) Immunostainings of MDCK cells for Wee1, together with α-catenin and Dapi, in an unperturbed high density monolayer and 1 h after induction of epithelial expansion by removal of the PDMS stencil in presence of the proteasome inhibitor MG132 (5µM).

(B) Immunostainings of dense MDCK monolayers, treated with the myosin phosphatase inhibitor Calyculin A (15 min; 10 ng/ml) or DMSO control, for Wee1 together with α-catenin and Dapi.

(C) Quantification of Wee1 immunofluorescence intensity per cell (normalized to the level of α-catenin intensity) in dense MDCK monolayers following treatment with DMSO (control) or the myosin phosphatase inhibitor Calyculin A (15 min; 10 ng/ml). n = 90 cells per condition. Data were pooled from 3 independent experiments. Black bars represent the mean and SD of the individual experiments. **P = 0.0085; ratio paired t-test.

(D) Immunostainings of dense control MDCK monolayers, and monolayers subjected to 30 min of 18% uniaxial stretch, for Wee1 together with α-catenin and Dapi.

(E) Immunostainings of MDCK cells for Cdk1 pY15, together with α-catenin and Dapi, in an unperturbed dense monolayer and 1 h after induction of epithelial expansion by removal of a PDMS stencil (indicated in grey).
(F) Immunostainings of dense control MDCK monolayers, and monolayers subjected to 30 min of 18% uniaxial stretch, for Cdk1 pY15 together with α-catenin and Dapi.
(G) Immunostainings of dense MDCK monolayers, treated with the myosin phosphatase inhibitor Calyculin A (15 min; 10 ng/ml) or DMSO control, for Cdk1 pY15 together with α-catenin and Dapi.
(H) Quantification of Cdk1 pY15 immunofluorescence intensity per cell (normalized to the level of α-catenin intensity) in dense MDCK monolayers following treatment with DMSO (control) or the myosin phosphatase inhibitor Calyculin A (15 min; 10 ng/ml). n = 90 cells per condition. Data were pooled from 3 independent experiments. Black bars represent the mean and SD of the individual experiments. ****P = 0.0001; ratio paired t-test.
All scale bars represent 10 μm.

Donker et al., Figure S6

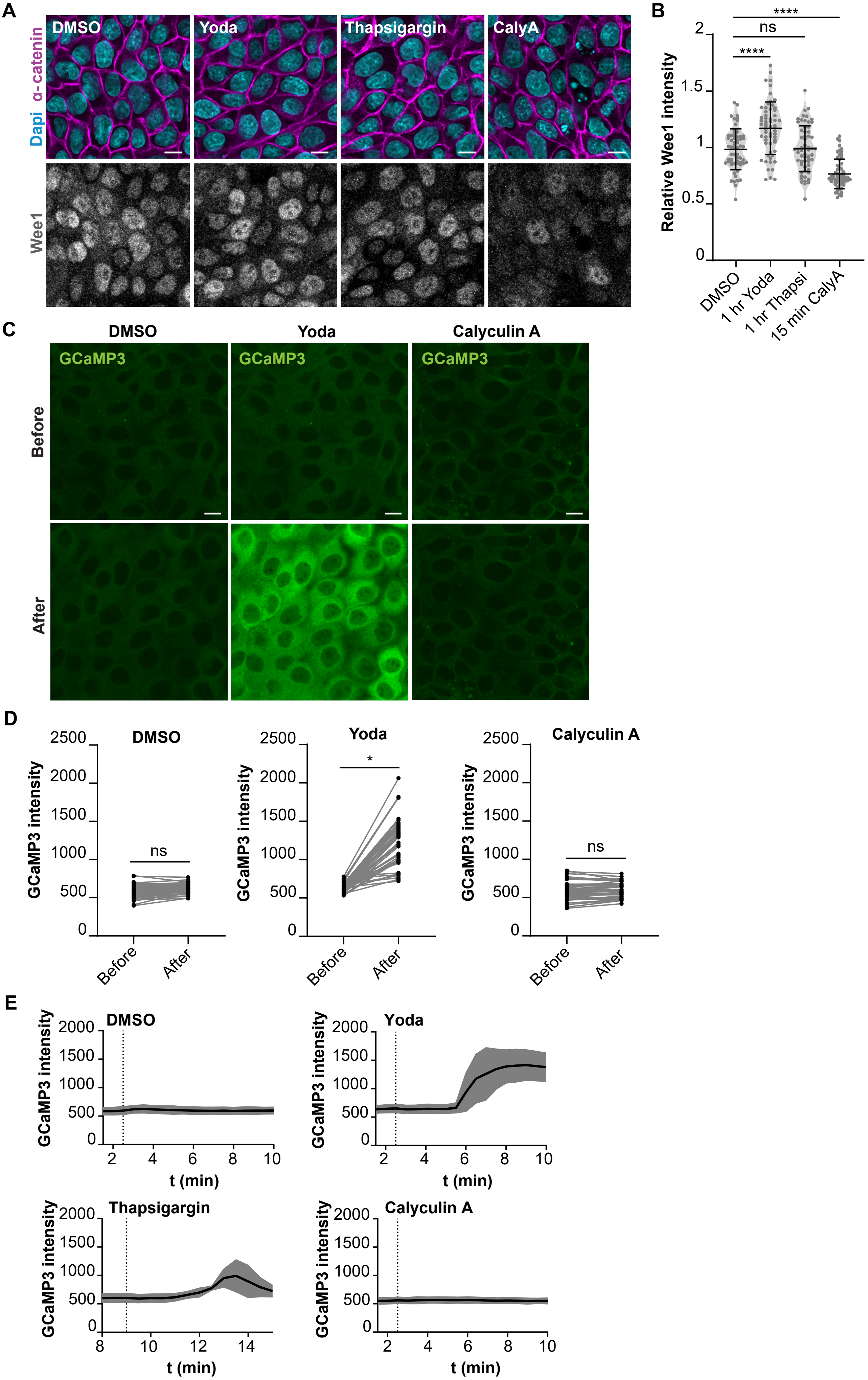

Supplementary Figure 6. Activation of Piezo1 and downstream calcium influx do not affect Wee1 protein levels, Related to Figure 6.

(A) Immunostainings of MDCK monolayers treated with DMSO, the Piezo1-selective agonist Yoda (1h; 10 μM), thapsigargin (Thapsi) (1h; 1 μM) to induce a calcium influx, or the myosin phosphatase inhibitor Calyculin A (15 min; 10 ng/ml), for Wee1 together with α-catenin and Dapi.

(B) Quantification of Wee1 immunofluorescence intensity per cell (normalized to the level of α-catenin intensity) in dense MDCK monolayers treated with DMSO, the Piezo1-selective agonist Yoda (1h; 10 μM), thapsigargin (Thapsi) (1h; 1 μM), or Calyculin A (15 min; 10 ng/ml). n = 60 cells per condition. Data were pooled from 2 independent experiments. Black bars represent the mean and SD. ****P <0.0001; ns = not significant; Mann-Whitney.

(C) Representative still images of MDCK cells stably expressing the GCaMP3 calcium reporter to monitor intracellular calcium levels (Tian et al., 2009), followed over time before and after treatment with DMSO, Yoda (10 μM) and Calyculin A (10 ng/ml). This shows that Calyculin A (which results in Wee1 downregulation, Figures S5B, S5C, S6A and S6B) does not induce a calcium influx, in contrast to Piezo1 activation by Yoda.

(D) Quantification of the GCaMP3 fluorescence signal (mean gray value) per cell in MDCK monolayers before and after treatment with DMSO, Yoda (10 μM) or Calyculin A (10 ng/ml). n = 45 cells per condition. Data were pooled from 3 independent experiments. *P = 0.026; ns = not significant; paired t-test.

(E) Representative traces of the fluorescence intensity (mean gray value) of MDCK GCaMP3 cells, followed over time before and after treatment with DMSO, Yoda (10 μM), thapsigargin (1 μM) or Calyculin A (10 ng/ml). The black line indicates the mean fluorescence signal, with the SD indicated in grey.

All scale bars represent 10 μm.
